# Molecular and morphological data reveal three new cryptic species of *Chiasmocleis* (Mehely 1904) (Anura, Microhylidae) endemic to the Atlantic Forest, Brazil

**DOI:** 10.7717/peerj.3005

**Published:** 2017-02-21

**Authors:** Mauricio C. Forlani, João F.R. Tonini, Carlos A.G. Cruz, Hussam Zaher, Rafael O. de Sá

**Affiliations:** 1 Department of Biology, University of Richmond, Richmond, VA, United States; 2 Museu de Zoologia, Universidade de São Paulo, São Paulo, Brazil; 3 Department of Biological Sciences, The George Washington University, Washington, D.C., United States; 4 Departamento de Vertebrados, Museu Nacional, Universidade Federal do Rio de Janeiro, Rio de Janeiro, Brazil

**Keywords:** Phylogeny, Molecular, Osteology, *Chiasmocleis*, Three new species, Atlantic Forest, Distribution

## Abstract

Three new cryptic species of *Chiasmocleis* from the Atlantic Forest of Brazil are described. Two of these species occur in the northeastern states of Sergipe and Bahia, whereas the third species is found in the southeastern state of São Paulo. The new species can be distinguished from other congeneric species by the molecular data, as evidenced in the phylogeny, and by a combination of morphological characters including: size, foot webbing, dermal spines, and coloration patterns. *Chiasmocleis* species differ in osteological traits, therefore we also provide an osteological description of each new species and comparsions with data reported for other species in the genus.

## Introduction

New World microhylids consist of two monophyletic subfamilies that are not closely related to each other (de Sá et al., 2012; Peloso et al., 2016). The diverse Gastrophryninae Wassersug and Pyburn, 1987 includes eleven genera and 70 species, whereas Otophryninae Fitzinger, 1843 consists of two genera and six species. *Chiasmocleis*, the largest radiation within gastrophrynines, are widely distributed throughout South America east of the Andes. Fifteen species are distributed throughout the Amazonian rainforest, twelve occur in the Atlantic Forest, and three are associated with open areas such as the Brazilian Cerrado and the Chaco of Bolivia and Paraguay (Caramaschi & Cruz, 2001; de Sá et al., 2012; Peloso et al., 2014). However, most species are known only from the type locality or have restricted distributions (e.g.,  *C. centralis*, *C. gnoma*, *C. sapiranga*, *C. atlantica*, *C. cordeiroi*, *C. crucis*, *C. haddadi*, *C. magnova*, *C. quilombola*, *C. mehelyi*).

*Chiasmocleis* exhibits sexual dimorphism associated with size, presence and distribution of dermal spines, color patterns, and degree of webbing (Miranda-Ribeiro, 1924; Bokermann, 1952; Carvalho, 1954; Cruz, Caramaschi & Izecksohn, 1997). Furthermore, foot webbing was used to identify two morphologically distinct “species-groups” of Atlantic Forest species (i.e., absent or vestigial webbing vs. well-developed webbing, Cruz, Caramaschi & Izecksohn, 1997). Although amount of webbing is useful to distinguish sympatric species (e.g.,  *C. schubarti* and *C. capixaba*), a recent molecular phylogeny showed that this character does not define monophyletic species-groups (de Sá et al., 2012).

Taxonomic revisions of *Chiasmocleis* populations distributed across the lowlands of the Atlantic Forest resulted in description of new species (e.g.,  *C. atlantica, C*. *capixaba*, and *C. lacrimae*) and taxonomical changes (e.g., revalidation of *C. leucosticta* (Boulenger 1888) and *C. schubarti* Bokermann, 1952 (Cruz, Caramaschi & Izecksohn, 1997)). More recently, a phylogeographic analysis of *C. lacrimae* and *C. capixaba* recovered high genetic diversity and low gene flow among populations (Tonini, Costa & Carnaval, 2013). Subsequent analyses, with an increased taxon and genetic sampling, recovered a new species, *C. quilombola* (Tonini, Forlani & de Sá, 2014); moreover, the authors suggested the presence of an additional undescribed species.

Biodiversity surveys along the Atlantic Forest have helped to understand cryptic diversity within *Chiasmocleis* endemic to the biome. Herein, we describe three new cryptic species found in isolated areas of Atlantic rainforest in the states of São Paulo, Bahia, and Sergipe, Brazil. Anuran systematics has extensively used osteological data; herein, we describe the osteology of the new species and compare it with the limited available data for *Chiasmocleis*.

## Material and Methods

Specimens used herein and comparative material, including type series, are deposited in the following collections: Coleção Célio F. B. Haddad, Departamento de Zoologia, Universidade Estadual Paulista, campus Rio Claro (CFBH); Museu de História Natural Capão da Imbuia, Curitiba (MHNCI); Museu Nacional, Rio de Janeiro (MNRJ); Museu de Zoologia, Universidade De São Paulo, São Paulo (MZUSP); Museu de Zoologia da Universidade Estadual de Santa Cruz, Santa Cruz (MZUESC); and Museu de Zoologia, Universidade Federal da Bahia, Salvador (UFBA). The specimens analyzed are listed in Appendix I. Gastrophryninae systematics and generic definitions follow de Sá et al. (2012); the former genus *Syncope* is currently placed under *Chiasmocleis* (Peloso et al., 2014).

The following measurements were adapted from Duellman (2001) and Peloso & Sturaro (2008) and taken with an electronic caliper under a stereomicroscope (to the nearest 0.1 mm): SVL (snout-vent length); HL (head length; from snout to angle of the jaw); HW (head width; between the angle of jaws); ED (eye diameter; between anterior and posterior corner of the eye); IOD (interorbital distance; distance between anterior corner of the eyes); IND (inter-narial distance); END (eye-nostril distance; from the anterior corner of the eye to the posterior margin of nostril); THL (thigh length; from the middle of the cloaca opening to the outer edge of the flexed knee); TBL (tibia length; from the outer edge of the flexed knee to the heel); FAL (forearm length); HDL (hand length; from the base of the thenar tubercle to the tip of the third finger); FL (foot length; from tibio-tarsal articulation to tip of fourth toe); 3FD (diameter of third finger disk); 4TD (diameter of fourth toe disk). Fingers and toes are numbered and abbreviated as follows: fingers I–IV = FI–IV, Toes I–V = TI–V.

A specimen from each of the new species was clear and double stained for osteological description (Appendix I). Terminology for osteology follows de Sá & Trueb (1991) and Trueb, Diaz & Blackburn (2011) except for the *manus* that follows Fabrezi & Alberch (1996). Drawings were made under a Leica stereomicroscope with a camera lucida attachment. Adult males were identified by the characteristic dark coloration of the gular region during the breeding season; sex of other specimens was done by dissection to identify sex organs.

The occipital fold in microhylids is variable (Wild, 1995) consisting of the following character states: (1) complete (distinct medially) or (2) incomplete (indistinct medially). However, for some species of *Chiasmocleis* the occipital fold was reported absent; however, it is usually present in large female and males specimens, e.g., *Chiasmocleis schubarti*, *C. avilapiresae*, and *C. leucosticta*. Furthermore, for other Gastrophyninae the character was described as: “…occipital fold complete, faint medially, more pronounced laterally…” (*Hamptoprhyne alios*, Wild, 1995) and “…postcephalic skin fold, immediately behind the eyes and extending and reaching laterally and posteriorly, to reach the insertion of the forelimb. …” (*Elachistocleis*, Lavilla, Vaira & Ferrari, 2003). We consider the character in the genus *Chiasmocleis* as an incomplete occipital fold.

Tissues were available for two of the three new species. Total genomic DNA was extracted from ethanol-preserved liver or muscle tissues using Qiagen DNeasy kit (Valencia, CA, USA). We used the genetic matrix presented in Tonini, Forlani & de Sá (2014), which included one of the new species; we also included samples of another new species as well as of *C. alagoana* and *C. shudikarensis*. Thus, the new dataset comprises samples of *C. mantiqueira*, *C. leucosticta*, *C. alagoana*, *C. cordeiroi*, *C. crucis*, *C. schubarti*, *C. capixaba*, *C. lacrimae*, *C. quilombola*, and the two *Chiasmocleis* species describe herein (Appendix I). These does not represent all species endemic of the Atlantic Forest (i.e., missing are *C. sapiranga*, *C. atlantica*, and *C. gnoma*); however, it includes all species that are sympatric or have the potential to occur in sympatry with the new species. The molecular markers used are 12S, 16S, ND2, and BDNF amplified using previously published primer sets and PCR profiles (de Sá et al., 2012; Tonini, Costa & Carnaval, 2013). Genbank accession numbers for sequences are provided in Appendix II. We grouped individuals by species and used the 16S data to calculate the *p*-uncorrected average genetic distance and standard errors.

The newly assembled data matrix was analyzed using the following procedure. First, we performed multi-sequence alignment and tree estimation in SATé-II (Liu et al., 2012). Second, we run PartitionFinder v1.1.1 (Lanfear et al., 2012) and find the best partition scheme and the respective substitution models according to Bayesian Information Criterion (BIC). Third, we used a concatenated matrix partitioned according to the results of PartitionFinder and estimated the phylogeny in BEAST v2.4.2 (Bouckaert et al., 2014). We unlinked the substitution model and linked clock model and trees across partitions using BEAUTI. We used Birth–death model as tree prior on the species tree, exponential distribution for speciation rate, relaxed clock model with lognormal distribution, and the mean clock rate and standard deviation were estimated along the analyses using a log normal distribution. We performed four independent runs of 100 million generations sampling the posterior distribution every 10,000th generation to yield a distribution of 10,000 trees in each run. Then, we combined results of the four runs using LogCombiner and check for convergence using Tracer v1.6 (Rambaut et al., 2014). Effective Sample Size (ESS) estimates higher than 200 were considered suitable. We discard the first 25% of the trees as burn-in using TreeAnnotator to generate a maximum credibility clade tree. We consider well-supported branches those with posterior probabilities equal or higher than 0.95.

In addition, we estimated the species tree using ASTRAL-II v4.10.11 (Mirarab et al., 2014; Mirarab & Warnow, 2015; Sayyari & Mirarab, 2016). ASTRAL is a coalescent species tree method that considers gene tree incongruence by modeling the process of incomplete lineage sorting under the multi-species coalescent model (Rannala & Yang, 2003).

We estimated the substitution model for each of the four genes separately using jModelTest 2 (Darriba et al., 2012; Guindon & Gascuel, 2003) and generated gene trees in BEAST. We used Yule model as tree prior on gene trees, a relaxed clock model with lognormal distribution, and the mean clock rate and standard deviation were estimated along the analyses using a log normal distribution. We run the analyses of each gene tree for 100 million generations and sampled every 10,000th generation. We check for convergence using similar procedure aforementioned for the concatenation analysis. Then, we randomly sampled 100 trees out of the posterior distribution of 10,000 trees using LogCombiner. These sample of 100 posteriors trees of each gene (i.e., 400 posteriors gene trees) was used to estimate the species tree. Then, the whole posterior distribution of trees (i.e., 40,000 posteriors gene trees) was used to score the branches.

Branch lengths in ASTRAL are in coalescent units and are prone to gene tree estimation error, which has been a source of criticism to species tree summary methods (Tonini et al., 2015), and may cause underestimation. Therefore, to accommodate gene tree estimation error we provided as input to ASTRAL a distribution of posterior trees. By doing this we tried to accommodate gene tree variation into the coalescent species tree summary analysis instead of providing a single Maximum Likelihood gene tree.

ASTRAL outputs the normalized quartet score, which represents the portion of gene tree quartets satisfied by the species tree. For instance, a score of 0.8 represents that 80% of the quartets observed in the gene trees are present in the species tree. Branch support values in ASTRAL measures the support for a quadri-partition (i.e., the four clusters around a branch). The local posterior probabilities are computed based on what percentage of quartets in the gene trees agree with a branch in the species tree, which is called quartet support (Sayyari & Mirarab, 2016). In addition, ASTRAL outputs alternatives local posterior probabilities for the main topology and one for each of the two alternative ones. The posterior of the three topologies adds up to one because ASTRAL assumes the four groups around the branch are correct and, therefore, there are only three possible alternatives (Sayyari & Mirarab, 2016). The measures of branch lengths, quartet support, and alternative local posterior probability have been demonstrated to have high precision in simulations and empirical data sets with different levels of incomplete lineage sorting (Sayyari & Mirarab, 2016). Analyses in BEAST and jModelTest were performed on CIPRES (Miller, Pfeiffer & Schwartz, 2010)] and trees were visualized and edited in FigTree.

The electronic version of this article in Portable Document Format (PDF) will represent a published work according to the International Commission on Zoological Nomenclature (ICZN), and hence the new names contained in the electronic version are effectively published under that Code from the electronic edition alone. This published work and the nomenclatural acts it contains have been registered in ZooBank, the online registration system for the ICZN. The ZooBank LSIDs (Life Science Identifiers) can be resolved and the associated information viewed through any standard web browser by appending the LSID to the prefix http://zoobank.org/. The LSID for this publication are: urn:lsid:zoobank.org:act:C25510A7-8031-4EC6-B966-29F8A95DE8F6; urn:lsid:zoobank.org:act:528CB36C-871C-4A94-B524-E9C6B05734FD; and urn:lsid:zoobank.org:act:5D2C0A91-9A1E-4379-A047-D4E967E69942. The online version of this work is archived and available from the following digital repositories: PeerJ, PubMed Central and CLOCKSS. Research was performed under University of Richmond Institutional Review Board, #IACUC 15-05-001.

The distribution of the three new species is restricted to the Atlantic Forest and we provide comparisons to diagnose the new species from other Atlantic Forest species.

## Results

**Figure 1:**
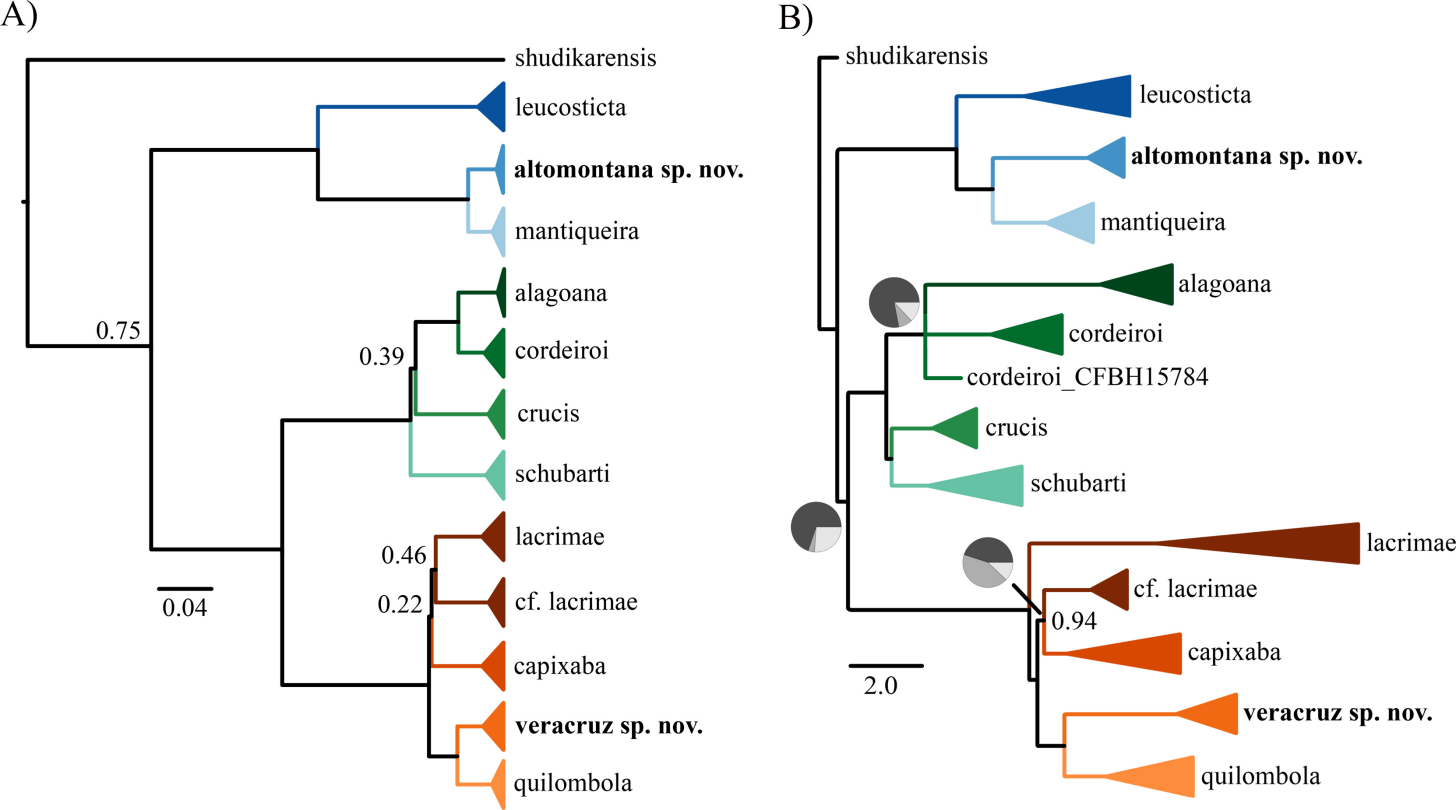
Phylogenetic tree. (A) Bayesian phylogenetic tree using concatenation and (B) coalescent species tree. In both phylogenies, only posterior probabilities lower than 0.95 are shown. Pie charts in (B) indicate the percentage of quartets in the gene trees that agree with the species tree.

In the BEAST analyses (both for concatenation and gene trees) all relevant parameters have ESS values higher than 200 and the analyses ran long enough to reach convergence. The molecular phylogenies using concatenation and species tree summary methods recovered monophyletic groups comprising individuals of described and undescribed species from the Atlantic Forest (Fig. 1, Supplemental Information). We work under the phylogenetic and the General Lineage Concept of Species (Hennig, 1966; De Queiroz, 1998; De Queiroz, 2007; Wheeler, 1999).

The normalized quartet score of the species tree was 0.81, representing that 81% of the quartets observed in the species tree are present in the gene trees. The concatenation (Fig. 1A) and species tree (Fig. 1B) recovered similar number of species but distinct interspecific relationships. The topological differences are in branches with weak support and/or incongruent among gene trees. For instance, we found differences among concatenation and species tree in the relationship of *C. crucis* and one sample of *C. cordeiroi*, which has distinct placement across alternative topologies of the species tree (Fig. 1B). However, it was recovered within the *C. cordeiroi* clade in the concatenated analysis (Fig. 1A).

Samples of *Chiasmocleis lacrimae* from a contact zone with *C. capixaba* were recovered as paraphyletic but with low posterior probability support of 0.46 (Fig. 1A) and 0.94 (Fig. 1B). We reported similar results previously (Tonini, Forlani & de Sá, 2014) and the phylogenetic relationship of these populations are still contentious.

The phylogenies show well-supported clades of allopatric populations that were not recognized as species, which we further examined for morphological differences. Herein, we describe three new cryptic species found in isolated areas of Atlantic rainforest in the states of São Paulo, Bahia, and Sergipe, Brasil.

### Description of new species

The three new cryptic species are assigned to the genus *Chiasmocleis* based on: (1) procoracoid cartilages and clavicles reduced, not reaching the glenoid region; (2) quadratojugal and maxilla not in contact; (3) alary process of premaxilla inclined slightly forward; (4) anterior vomers present; (5) posterior vomer and neopalatines absent; and (6) four distinctive fingers and five toes (Parker, 1934; Carvalho, 1954; these characters variably apply to the clade of *Chiasmocleis* species formely placed in the genus *Syncope*).

**Table:** 

**Figure 2:**
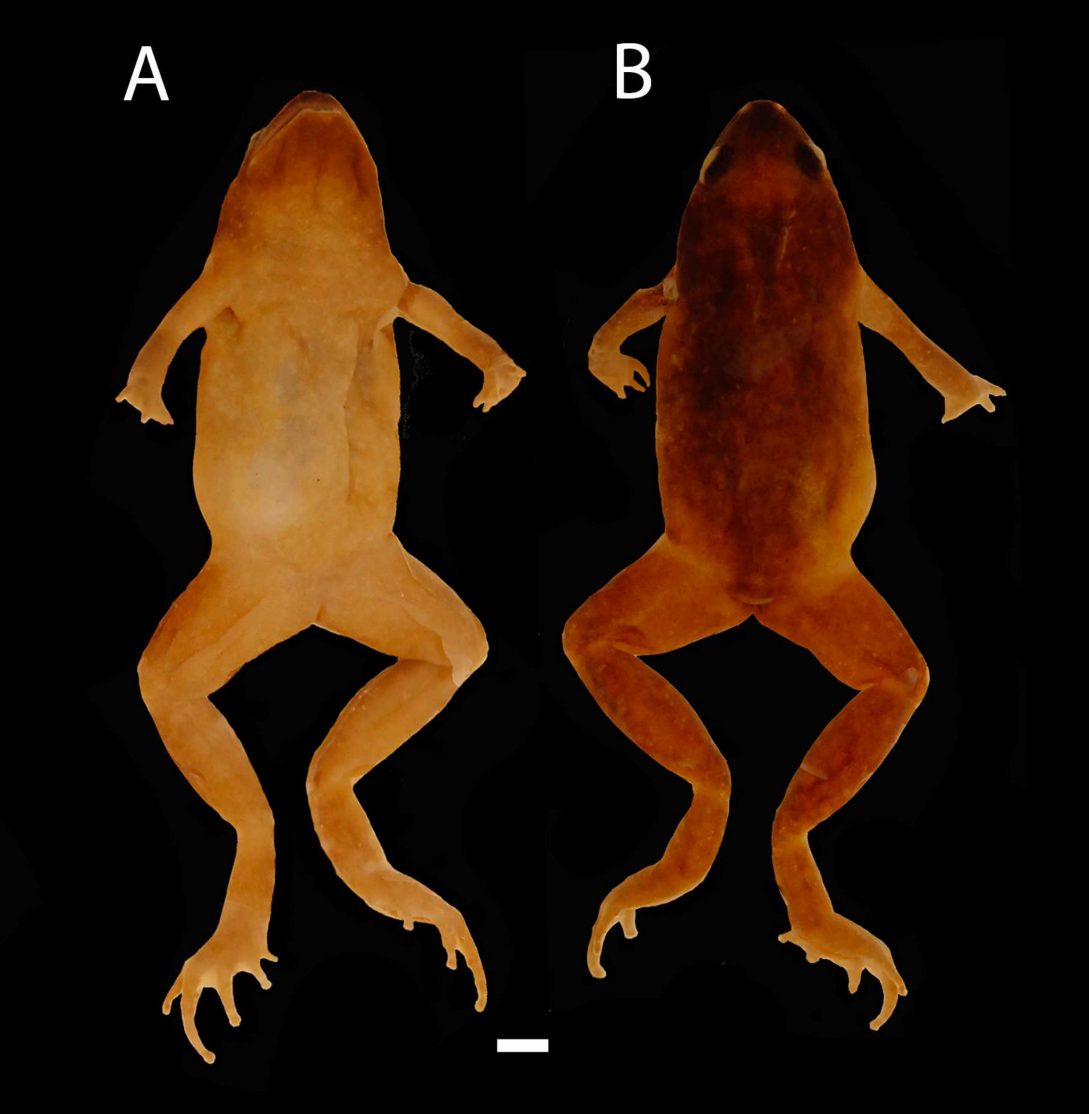
Images of Holotype of *Chiasmocleis migueli*. Holotype of *Chiasmocleis migueli* sp. nov., MZUSP 114583, male. (A) Ventral and (B) Dorsal views.

***Chiasmocleis migueli*** **sp. nov.** (Fig. 2)

**Holotype.** MZUSP 114583, adult male, collected at the Municipality of Santa Luzia do Itanhy, State of Sergipe, Brazil (11°20′55″S; 37°26′44″W), on March 2001, collected by STP Amarante and MT Tavares.

**Paratypes.** MZUSP 114582, 114584 (both young females), and MZUSP 114585 (juvenile). All collected at type locality on March 2001 by STP. Amarante and MT Tavares.

**Diagnosis.** A medium-size species of *Chiasmocleis* (male SVL 20.0 mm; no available data for adult females), diagnosed by the following combination of characters: (1) body slender; (2) snout rounded in lateral and dorsal views; (3) first finger short in male; (4) fingers slightly fringed, except the first one; (5) fingers not webbed; (6) toes fringed; (7) toes not webbed in females, webbed in male; (8) fingers and toes with small lateral dermal spines in male, absent in females; (9) incomplete occipital fold; (10) vocal slits present in males; (11) dorsal surface of the body with small, uniformly distributed dermal spines in male, absent in females; (12) dorsal coloration brown; (13) background of ventral body surface pale cream with a fine and homogeneous marbled pattern.

**Figure 3:**
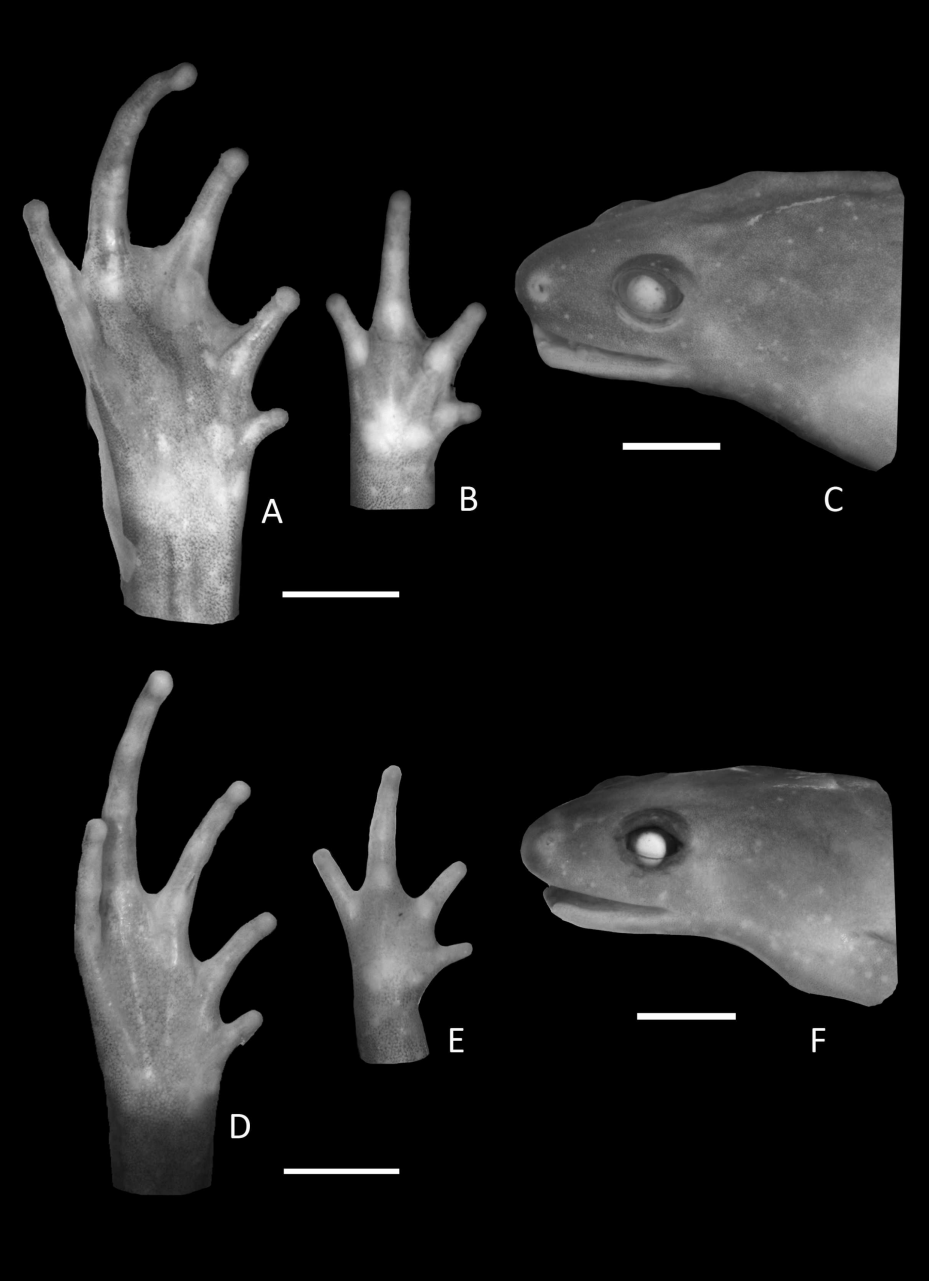
Foot, hand, and lateral view details of Holotype of *Chiasmocleis migueli*. Ventral view of the foot, hand, and lateral profile of the head of *Chiasmocleis migueli* sp. nov.; (A–C) Holotype MZUSP 114583, male; (D–E) Paratype MZUSP 114584, female.

**Description of Holotype.** Body slender, slightly ovoid (Fig. 2); head triangular broader than long; snout, short snout tip rounded in dorsal and lateral views (Fig. 3); nostrils located at the tip of snout, not protuberant, directed laterally; internostril distance smaller than eye–nostril distance and slightly smaller than eye diameter; canthus rostralis weakly defined; loreal region slightly concave; lips not flared; eyes small only slightly protruding; upper eyelid width one third of the interorbital space; interorbital area flat; incomplete occipital fold, weakly evident only laterally behind the eyes; tympanum indistinct; upper jaw prognathus; mandible truncated with a trilobed anterior margin; tongue large, ovoid, posteriorly unnotched; premaxillae maxillae and vomerine teeth absent; choanae small, rounded, widely separated, positioned anterolaterally to eye; a single smooth and long palatal ridge present; vocal slit present, vocal sac small and subgular.

Arms slender, lacking tubercles and crests on forearm. Hand not webbed; fingers lacking disks and slightly fringed, except the first one, with lateral dermal spines; finger lengths I < II < IV < III; thumb without nuptial asperities; subarticular tubercles well developed, rounded; supernumerary tubercles absent; adpressed FI do not reach the proximal subarticular tubercle of FII; adpressed FII do not reach the distal tubercle of FIII; adpressed FIV reach the proximal margin of the distal tubercle in FIII; thenar tubercle well developed, ovoid and at the base of finger I; palmar tubercle large, divided into a round and an elongate parts, (Fig. 3). Legs short, moderately robust; combined thigh and tibia lengths approximately 87% of snout-vent length; foot length approximately 40% of snout-vent length; knees and heels without tubercles; tibial and tarsal ridges absent. Foot webbed webbing does not extend beyond the second subarticular tubercle of toes V and IV; toes slightly fringed; toes with small disks; toes with few small lateral dermal spines in males; subarticular tubercles well-developed, ovoid; supernumerary tubercles absent; an oval inner, but no outer, metatarsal tubercle (Fig. 3). Adpressed TI does not reach subarticular tubercle of TII; adpressed TV contact the base of the middle subarticular tubercle of TIV; toe lengths I < II < V < III < IV; thigh length slightly longer than tibia length.

Ventral surface of skin smooth; dorsal surfaces of body and limbs with small, uniformly distributed dermal spines in males, absent in females (Fig. 2). Cloacal opening not modified, lacking para-cloacal tubercles and glands

**Coloration in preservative of the holotype.** Dorsum uniformly dark brown; dorsal surface of limbs marbled dark brown and pale cream; hands and feet brown; palm of hands and foot pale cream; middorsal dorsal line absent; medial line on posterior thighs absent; ventral surfaces homogeneously and finely marbled in pale brown and cream; male throat infuscated.

**Measurements of Holotype.** SVL 20.0; HL 3.9; HW 5.3; ED 1.6; IOD 3.0; IND 1.2; END 1.6; THL 8.7; TBL 8.5; FL 7.9, HDL 4.4; FAL 5.2; 3FD 0.3; 4TD 0.5.

**Variation in the type series**. Among the type series, a whitish longitudinal mid-dorsal line is present in one female (MZUSP 114584), absent in all other type specimens. This female also has irregular white blotches on outer surface of arms and forearms. The belly patterns of the type series show a few distinct white spots, absent in the holotype. The two young females lack foot webbing, present in the holotype (male, Fig. 3). The palatal ridge is not visible in paratype MZUSP 114585.

**Comparisons with other species**. There are no known *Chiasmocleis* species that occur in sympatry with *C. migueli* sp. nov.; the geographical closest species is *C. alagoana*. The new species can be distinguished by its small size (*C. alagoana* SVL 22.7–23.4 mm, Cruz, Caramaschi & Freire, 1999), males with webbed feet (webbing absent in *C. alagoana*), and belly pattern finely marbled in pale brown and cream (roughly marbled in dark brown and pale cream in *C. alagoana*).

*Chiasmocleis migueli* sp. nov. can be distinguished from other Atlantic Forest species by: males having webbed feet (absent or small web in *C. atlantica*, *C. lacrimae*, *C. gnoma*, *C. schubarti*, *C. quilombola*, and *C. veracruz* sp. nov., Cruz, Caramaschi & Izecksohn, 1997; Cruz, Caramaschi & Freire, 1999; Canedo, Dixo & Pombal, 2004; Tonini, Forlani & de Sá, 2014; present study); foot webbing reaching but not extending beyond the first and second subarticular tubercles between toes III and V (extending beyond the tubercles in *C. cordeiroi*, *C. leucosticta*, *C. mantiqueira*, *C. sapiranga*, and *C. altomontana* sp. nov.); females lack of webbing on feet (present in *C. cordeiroi*, *C. leucosticta*, *C. mantiqueira*, *C. sapiranga*, and *C. altomontana* sp. nov., Cruz, Caramaschi & Izecksohn, 1997; Caramaschi & Pimenta, 2003; Cruz, Caramaschi & Napoli, 2007; Cruz, Feio & Cassini, 2007; present study); male fingers without webbing (present in *C. altomontana* sp. nov., *C. leucosticta*, and *C. mantiqueira*, Cruz, Caramaschi & Izecksohn, 1997; Cruz, Caramaschi & Napoli, 2007; present study); and males third finger not swollen (swollen in *C. capixaba*, *C. cordeiroi, C*. * mantiqueira, C*. *leucosticta,* and *C. sapiranga*); belly pattern finely marbled in pale brown and cream, without distinct dark or white spots or dots (belly pattern: roughly marbled in dark brown and pale cream in *C. atlantica*, *C. crucis*, *C. cordeiroi*, *C. leucosticta*, *C. sapiranga,* and *C. lacrimae*, Cruz, Caramaschi & Izecksohn, 1997; Cruz, Caramaschi & Freire, 1999; Caramaschi & Pimenta, 2003; Cruz, Caramaschi & Napoli, 2007; Cruz, Feio & Cassini, 2007; brown with large cream blotches in *C. gnoma*, Canedo, Dixo & Pombal, 2004; light cream in *C. quilombola*, Tonini, Forlani & de Sá, 2014; cream or white with few scattered dark brown blotches in *C. mantiqueira* and *C. altomontana* sp. nov.); infuscated throat (not infuscated in *C. mantiqueira* and *C. altomontana* sp. nov.). Male size in *C. migueli* is SVL 20.0 mm (more than 22 mm in *C. alagoana*, *C. atlantica*, and *C. lacrimae*, Cruz, Caramaschi & Izecksohn, 1997; Cruz, Caramaschi & Freire, 1999; less than 18 mm in *C. altomontana* sp. nov., *C. capixaba*, *C. lacrimae*, *C. gnoma, C. mantiqueira, C*. *quilombola*, and *C. veracruz* sp. nov., Cruz, Caramaschi & Izecksohn, 1997; Canedo, Dixo & Pombal, 2004; Cruz, Feio & Cassini, 2007; Tonini, Forlani & de Sá, 2014 present study).

**Figure 4:**
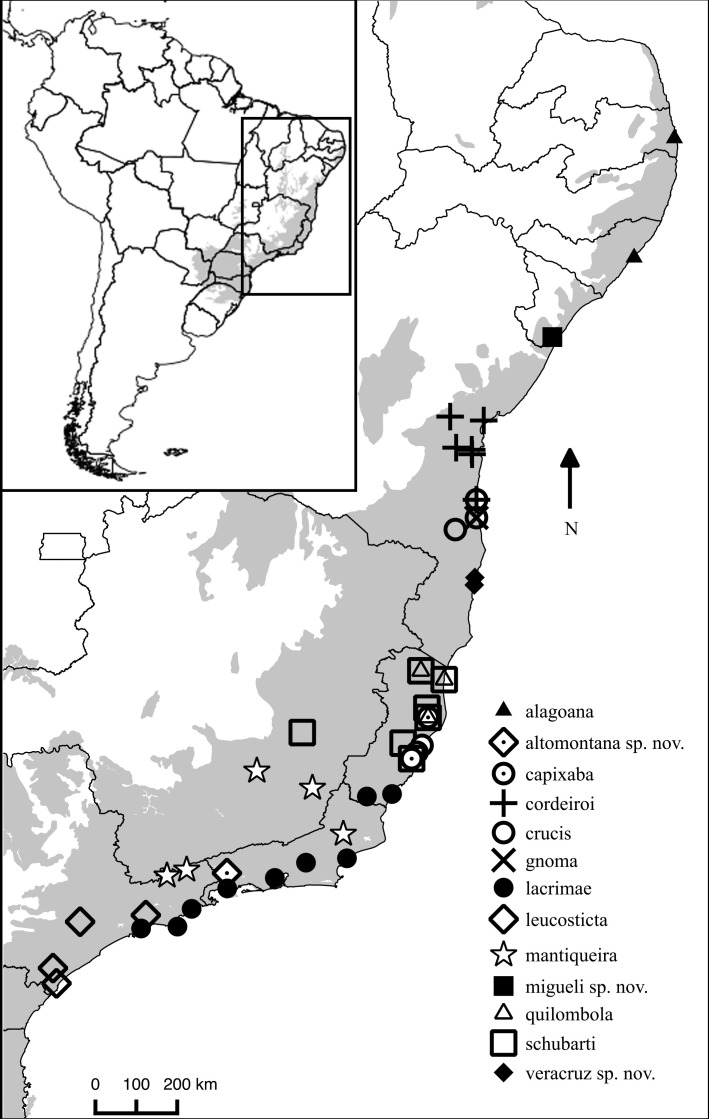
Distribution map of *Chiasmocleis* species in Atlantic Forest. Geographic distribution of *Chiasmocleis* in the Atlantic Forest, including those of the three new species. Grey area represents the limits of the Atlantic Forest.

**Distribution.** *Chiasmocleis migueli* sp. nov. is known only from the type locality, Santa Luzia do Itanhy, State of Sergipe, Brazil (Fig. 4), which is located at the south margin of the São Francisco river. *Chiasmocleis alagoana* occurs on the north margin of the São Francisco river and the type locality (and only known locality) of *C. sapiranga* is 230 km south of the type locality of *C. migueli*. Although most *Chiasmocleis* species in the Atlantic Forest are forest dependent (Cruz, Caramaschi & Freire, 1999; Caramaschi & Pimenta, 2003; Faria et al., 2007; Camurugi et al., 2010; Dixo & Metzger, 2010), northeastern species can also be found in disturbed forest at lower densities (Caramaschi & Pimenta, 2003; Canedo, Dixo & Pombal, 2004; Camurugi et al., 2010). The survivorship of populations of *C. migueli* is worrisome given that forest fragments in the State of Sergipe, and surrounded areas, are under significant deforestation (Landium & Fonseca, 2007).

**Etymology.** The specific epithet is to honor Dr. Miguel Trefaut Rodrigues, Universidade São Paulo, São Paulo, Brazil, for his extensive contributions, both in research and education, to South American herpetology, Dr. Rodrigues has an extensive fieldwork in Brazil, particularly in the Atlantic Forest; two of the new species describe herein resulted from his fieldwork program.

**Remarks.** Dermal spines are conspicuous structures during the breeding season in adults. The two young females examined do not have dermal spines; if present in the adult females, we anticipate they will occur around the cloacal region; this character as well as other information (e.g., SVL) awaits confirmation for adult females. The holotype shows evidence of molting (i.e., loose skin), after molting the dermal spines are not visible.

**Figure 5:**
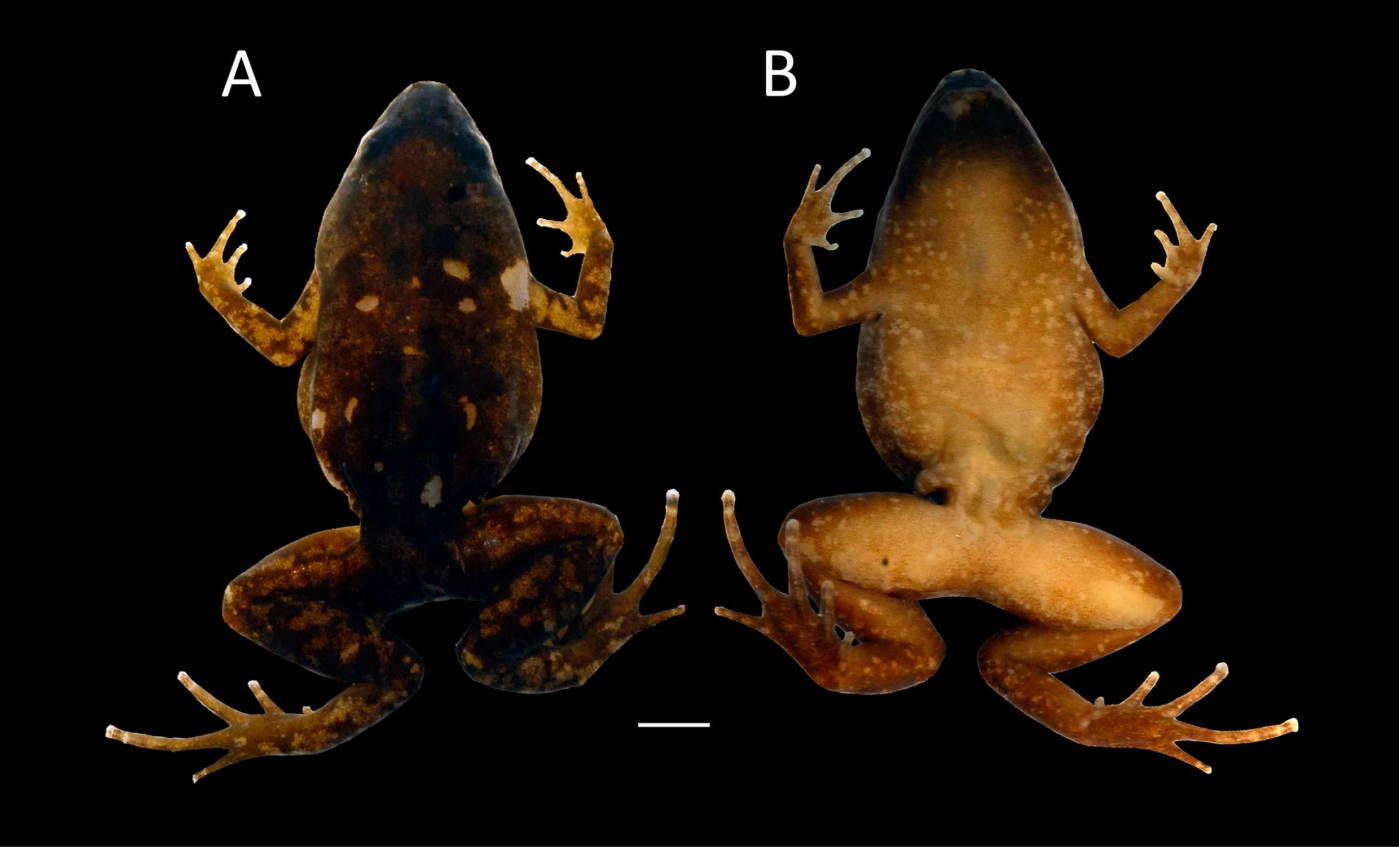
Holotype of *Chiasmocleis veracruz*. Holotype of *Chiasmocleis veracruz* sp. nov. MZUSP 149588, male. (A) Dorsal and (B) ventral views.

**Table:** 

***Chiasmocleis veracruz*** **sp. nov.** (Fig. 5)

**Holotype.** MZUSP 149588 (Field Number MTR13545), adult male, collected at Fazenda Nova Alegria, Municipality of Trancoso, State of Bahia, Brazil (16°32′11.76″S; 39°07′28.92″W), on March 2007 by MT Rodrigues.

**Paratypes.** MZUSP 149585-86, 149589-91, 149593, 149597 (Field Number MTR 13489, 13495, 13546-48, 13590, 13565), males; MZUSP 149583-84, 149592, 149594-95 (Field Number MTR 13465-66, 13549, 13579-80), females; MZUSP 149587, 149596 juvenil (Field Number MTR 13544, 13589) all collected with the holotype.

**Diagnosis.** A small-size species of *Chiasmocleis* (males *χ* = 15.3 mm; female SVL 16.7 mm, Table 1), diagnosed by the following characters: (1) body slender; (2) snout short, rounded in lateral and dorsal views; (3) fingers slightly fringed, not webbed; (4) toes fringed, without webbing in males and females; (5) fingers and toes with tiny small lateral dermal spines in males, absent in females; (6) body dorsal surface with few small dermal spines in males and around the cloaca in females; (7) vocal slits present in males; (8) incomplete occipital fold; (9) combined thigh and tibia lengths about 81.5% of snout-vent length; (10) foot length about 45% of snout-vent length; (11) dorsal coloration brown; (12) venter pale brown speckled with cream spots, more evident on the edges and throat; and (13) male throat infuscate.

**Table 1:** Summary statistics of *Chiasmocleis veracruz* sp. nov.

Measurements	Male ( *N* = 8)			Female ( *N* = 5)		
	Range	$\overline{X}$	SD	Range	$\overline{X}$	SD
SVL	13.8–16.3	15.3	0.7	16.3–17.4	16.7	0.4
HL	3.1–3.8	3.4	0.2	3.4–3.9	3.6	0.1
HW	4.0–4.6	4.2	0.2	4.5–4.6	4.4	0.0
ED	1.0–1.2	1.1	0.0	0.9–1.5	1.1	0.2
IOD	2.3–2.7	2.4	0.1	2.4–2.7	2.6	0.1
IND	0.8–1.1	0.9	0.0	0.8–1.2	0.9	0.1
END	1.1–1.6	1.3	0.1	1.4–1.6	1.5	0.0
THL	5.6–6.7	6.0	0.4	5.9–6.9	6.5	0.3
TBL	5.7–6.6	6.2	0.2	6.2–7.0	6.6	0.3
FL	5.7–6.8	6.5	0.3	6.9–7.7	7.1	0.3
HDL	3.2–3.7	3.4	0.1	3.6–4.2	3.8	0.2
3FD	0.3–0.3	0.3	0.0	0.3–0.3	0.3	0.0
4TD	0.4–0.5	0.4	0.0	0.3–0.5	0.4	0.0
FAL	2.9–3.6	3.1	0.2	3.1–3.6	3.2	0.2

**Notes.**

N number of specimens X mean SD standard deviation

**Figure 6:**
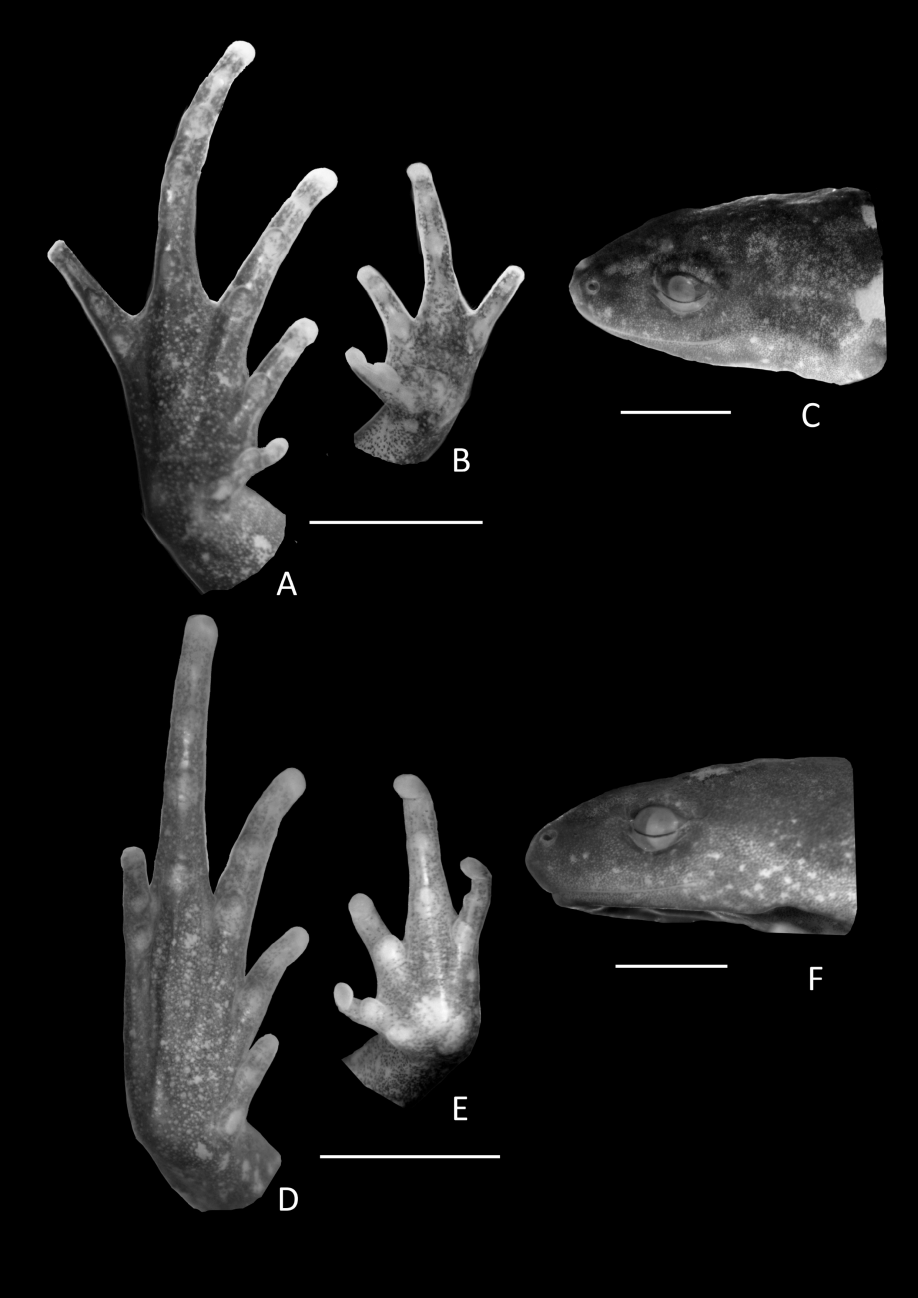
Holotype details of *Chiasmocleis veracruz*. Ventral view of the hand, foot, and lateral profile of the head of *Chiasmocleis veracruz* sp. nov.; (A–C) Holotype MZUSP 149588, male; (D–E) Paratype MZUSP 13465, female.

**Description of Holotype**. Body slender, slightly ovoid (Fig. 5); head triangular shape, broader than long; snout short, round in dorsal and lateral views (Fig. 6); nostrils located at the tip of snout, not protuberant directed laterally; internostril distance smaller than eye–nostril distance and slightly smaller than eye diameter; canthus rostralis poorly defined; loreal region slightly convex; lips not flared; eyes small, slightly protruding; upper eyelid not well defined; interorbital area flat; occipital fold incomplete; indistinct tympanum; upper jaw prognathus; mandible truncated with a trilobed anterior margin; tongue large, elongated, posteriorly unnotched; premaxillae, maxillae, and vomerine teeth absent; choanae small, rounded, widely separated, positioned anterolaterally to eye; two smooth palatal ridges anterior palatal ridge shorter and more distinct than posterior one; vocal slit present; vocal sac small and subgular.

Arms slender, lacking tubercles and crests on forearm Hands not webbed (Fig. 6); fingers lacking disks, slightly fringed, and with very small lateral dermal spines; finger lengths I < II < IV < III; thumb without nuptial asperities; subarticular tubercles well developed, rounded; subarticular tubercles on proximal phalanges larger than others; supernumerary tubercles absent; adpressed FI reaches the middle of the proximal subarticular tubercle in FII; adpressed FII reaches the base of the distal tubercle in FIII; adpressed FIV reaches the base of distal tubercle in FIII; thenar tubercle well developed, ovoid, at the base of finger I; palmar tubercle large, divided into two parts one rounded and the other elongate. Legs short moderately robust; knee and heel without tubercles; tibial and tarsal ridges absent. Foot not webbed (Fig. 6); toes slightly fringed; small toe disks; subarticular tubercles well developed, ovoid; supernumerary tubercles absent; an oval inner, but no outer, metatarsal tubercle. Toe lengths I < II < V < III < IV; adpressed TI does not reach subarticular tubercle of TII; adpressed TV reaches the base of the middle subarticular tubercle of TIV; toes with few very small lateral dermal spines; thigh length slightly shorter than tibia length.

Dorsal surfaces of body and limbs with scarcely and small dermal spines. Cloacal opening lacks para-cloacal tubercles and glands.

**Figure 7:**
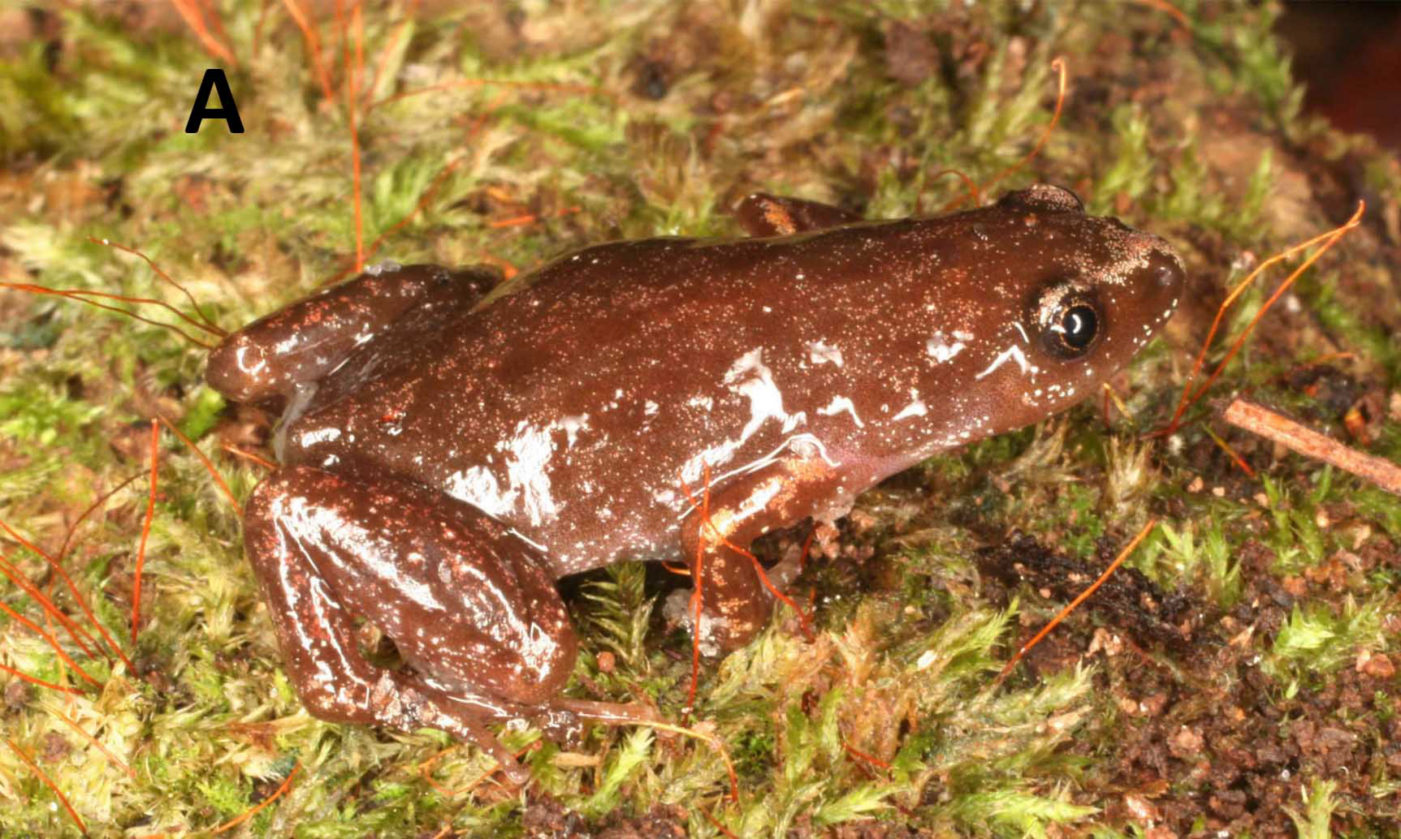
Live specimens. Live specimen of *Chiasmocleis veracruz* sp. nov.

**Coloration in preservative of the holotype.** Dorsum uniformly dark brown; dorsal surface of limbs marbled dark brown and pale cream; dorsal arms with cream blotches blotches darker on legs; dorsal surface of hands and feet brown; palm of hands marbled brown and pale cream, foot dark brown; venter pale brown speckled with cream spots, more evident on the edges and throat; throat blackish (Fig. 5). Ventral surface of thigh pale cream with brown speckled, more densely grouped on the edges; ventral surfaces of tibia and tarsus finely marbled in pale brown and cream, lighter than the dorsal surface. Middorsal line and line on posterior surface of thighs absent. We did not examine coloration of live specimens (photo of alive specimen courtesy of MT Rodrigues; Fig. 7).

**Measurements of Holotype**. SVL 15.1; HL 3.4; HW 4.1; ED 1.1; IOD 2.4; IND 1.0; END 1.4; THL 5.9; TBL 6.4; FL 6.8, HDL 3.4; FAL 3.0; 3FD 0.5; 4TD 0.4.

**Variation in the type series.** A summary of measurements is provided in Table 1. The types series agree with the coloration of the holotype, except for cream blotches found on the dorsal surface arms and legs, which can be absent or more evident in some specimens as well as a dark brown ventral pattern. Ventrally, very thin and whitish lines running from the arms to the midline may be present. Small dermal spines on the dorsum can be absent in males and females; dermal spines on fingers and toes always absent in females; the occipital fold varied from indistinct to weakly visible laterally. The type series show some small variation on the length of fingers in females: adpressed FI reaches the middle or distal edge of the proximal subarticular tubercle of FII; adpressed FII reaches the base or middle of distal tubercle in FIII; adpressed FIV reaches the base or middle of distal tubercle in FIII. The combined mean thigh and tibia length represents approximately 80.2% of mean snout-vent length in males and 79.2% in females; foot length approximately 42.8% of snout-vent length in males and females. The type series agree with the coloration of the holotype, except for cream blotches present in the dorsal surface of arms and legs, which can be absent or more evident in some specimens, and a dark brown ventral pattern.

**Comparisons with other species**. The following species occur along or close to the distribution of *Chiasmocleis veracruz* sp. nov.: *C. capixaba, C*. *quilombola, C*. *cordeiroi, C*. *crucis*, *C. gnoma,* and *C. sapiranga*. The new species can be distinguished from those species by: males lacking foot webbing (foot webbing present in *C. capixaba, C*. *cordeiroi*, *C. crucis*, and *C. sapiranga*, small webbing in *C. quilombola*, Cruz, Caramaschi & Izecksohn, 1997; Caramaschi & Pimenta, 2003; Cruz, Caramaschi & Napoli, 2007; Tonini, Forlani & de Sá, 2014); belly pattern pale brown speckled with cream spots, more evident on the edges and throat (belly pattern: roughly marble in dark brown and pale cream in *C. cordeiroi, C*. * crucis,* and *C. sapiranga*, Cruz, Caramaschi & Izecksohn, 1997; Caramaschi & Pimenta, 2003; Cruz, Caramaschi & Napoli, 2007; ligth cream colored in *C. quilombola,* Tonini, Forlani & de Sá, 2014; brown with large cream blotches in *C. gnoma*, Canedo, Dixo & Pombal, 2004); males fingers with poorly developed fringes (fingers well fringed in *C. cordeiroi* and *C. sapiranga*, Caramaschi & Pimenta, 2003); males third finger not swollen (males third finger swollen in *C. cordeiroi* and *C. sapiranga*, Caramaschi & Pimenta, 2003; Cruz, Caramaschi & Napoli, 2007); dermal spines small and scarce (dermal spines abundant and larger in *C. capixaba, C*. *cordeiroi*, *C. crucis*, and *C. sapiranga*); overall head shape with rounded snout and not prominent eyes (short truncate snout with prominent eyes in *C. gnoma*, Canedo, Dixo & Pombal, 2004). Furthermore, the new species is larger than *C. gnoma* (male *χ* = 13.9 mm; female *χ* = 15.9 mm, Canedo, Dixo & Pombal, 2004) and *C. quilombola* (male *χ* = 14.0 mm; female *χ* = 17.1 mm, Tonini, Forlani & de Sá, 2014) and smaller than *C. cordeiroi* (male *χ* = 21.5 mm; female *χ* = 25.9), *C. crucis* (male *χ* = 20.6 mm), and *C. sapiranga* (male *χ* = 19.9 mm; female *χ* = 23.5, Cruz, Caramaschi & Napoli, 2007).

The new species is morphologically similar to *Chiasmocleis quilombola* and *C. lacrimae*; however, molecular phylogenetic analyses consistly recovered them as evolutionary independent lineages. Furthermore, *C. veracruz* sp. nov., *C. quilombola*, and *C. lacrimae* are allopatrically distributed. Advertisment call is a trait likely to show variation due to sexual and natural selection; therefore they are potentially useful to differentiate cryptic species. Although advertisment calls of *C. quilombola* and *C. veracruz* sp. nov. are not yet available, *C. veracruz* sp. nov. differs from *C. quilombola* and *C. lacrimae* by having a pale cream belly pattern without dark spots or blotches (*C. lacrimae* roughly marbled in dark brown and pale cream; light brown and cream marbled pattern in *C. quilombola*); less robust arms and legs (robust arms and legs in *C. lacrimae*); and small and scarse dermal spines in males (dermal spines larger and abundant in *C. lacrimae*, small and abundant in *C. quilombola*).

In addition, *Chiasmocleis veracruz* sp. nov. can be distinguished from other Atlantic Forest species by: males and females lacked webbed feet (males and females with webbed feet in *C. leucosticta*, *C. mantiqueira*, and *C. altomontana* sp. nov., Cruz, Caramaschi & Izecksohn, 1997; Cruz, Feio & Cassini, 2007; present study; males with webbed feet in *C. capixaba* and *C. migueli* sp. nov., Cruz, Caramaschi & Izecksohn, 1997); belly pattern pale cream without dark spots or blotches (belly pattern roughly marbled in dark brown and pale cream in *C. alagoana*, *C. altomontana* sp. nov., *C. atlantica, C*. *leucosticta*, *C. mantiqueira*, and *C. schubarti*, Cruz, Caramaschi & Izecksohn, 1997; Cruz, Caramaschi & Freire, 1999; Cruz, Feio & Cassini, 2007, see below); males third finger not swollen (males third finger swollen in *C. altomontana* sp. nov., *C. leucosticta,* and *C*. *mantiqueira*, Cruz, Feio & Cassini, 2007); infuscated throat (throat not infuscated in *C. mantiqueira* and *C. altomontana* sp. nov., Cruz, Feio & Cassini, 2007).

*Chiasmocleis veracruz* also can be recognized by its smaller size (male SVL *χ* = 15.3 mm, females SVL *χ* = 16.7 mm) compare to *C. alagoana* (male SVL *χ* = 22.7–23.4 mm; female SVL *χ* = 26.8 mm, Cruz, Caramaschi & Freire, 1999), *C*.* atlantica* (male SVL *χ* = 23.1 mm, female SVL *χ* = 30.6 mm, Cruz, Caramaschi & Izecksohn, 1997), *C. altomontana* sp. nov. (male SVL *χ* = 17.2 mm; female SVL *χ* = 20.2 mm see below), *C. leucosticta* (male SVL *χ* = 20.0 mm; female SVL *χ* = 23.6 mm, Cruz, Caramaschi & Izecksohn, 1997), *C. mantiqueira* (male SVL *χ* = 16.5 mm; female SVL *χ* = 21.5 mm, Cruz, Feio & Cassini, 2007), *C. migueli* (male SVL *χ* = 20.0 mm; female SVL *χ* = 18.1 mm), *C. schubarti* (male SVL *χ* = 23.5 mm; female SVL *χ* = 28.7, Cruz, Caramaschi & Izecksohn, 1997), and *C. lacrimae* (male SVL *χ* = 17.1 mm; female SVL *χ* = 20.2 mm, Cruz, Caramaschi & Izecksohn, 1997).

**Distribution.** *Chiasmocleis veracruz* sp. nov. is restricted to southern region of the State of Bahia on the south margin of the Jequitinhonha river. This species is known from three sites in the Municipalities of Trancoso, Porto Seguro, and Una (Fig. 4). Two of those sites are preserved natural areas, Estação Ecológica de Vera Cruz (Veracel), Municipality of Porto Seguro, and Estação Ecológica de Una (EEU), Municipality of Una, Bahia, Brazil.

**Etymology.** The name refers to the first name “Terra da Vera Cruz” given to the “new land” of current Brazil by the Portugueses in 1500.

Scriber Pero Vaz de Caminha described in a letter (“A Carta de Pero Vaz de Caminha,” Caminha, 1500) the arrival of the Portuguese to this region (italic and bold fonts added to original text) *“…e neeste dia* [note: April 22] *a oras de bespera ouuemos vista de terra .s., primeiramente d huű gramde monte muy alto e rredondo e doutras serras mais baixas ao sul dele e de terra chaã com grandes aruoredos, ao qual monte alto o capitam pos nome o monte pascoal e aa tera a* ***tera da Vera cruz****...deste porto seguro da vosa* ***jlha da vera cruz*** *oje sesta feira prim.*^*o*^ *dia de mayo de 1500.”* [letter was sent on May 1st, 1500] [Translation: “....On this day [note: April 22], on the eve hours, we heard sight of land! First of a large mountain, very high and rounded, and other lower mountains, and to the south flat lands with large trees, the captain named the high mountain Monte Pascoal and the land as the **land of Vera Cruz** ... from this safe harbor of your **island of Vera Cruz**, today friday, first day of May of 1500] [letter was sent on May 1st, 1500]. The type locality of *C. veracruz* sp. nov. corresponds to the region where the ship docked in 1500. The name is used in apposition.

**Remarks.** The populations of *Chiasmocleis* from the Municipality of Porto Seguro and Una were previously referred as *C. carvalhoi* (Pimenta, Cruz & Dixo, 2002) and subquently assigned to *C. lacrimae* (Peloso et al., 2014). Herein, we assigned those populations to *C. veracruz* sp. nov. In addition specimens of *Chiasmocleis veracruz* sp. nov. were reported as *Chiasmocleis* sp. in the description of *C. quilombola* (Tonini, Forlani & de Sá, 2014).

**Table:** 

**Figure 8:**
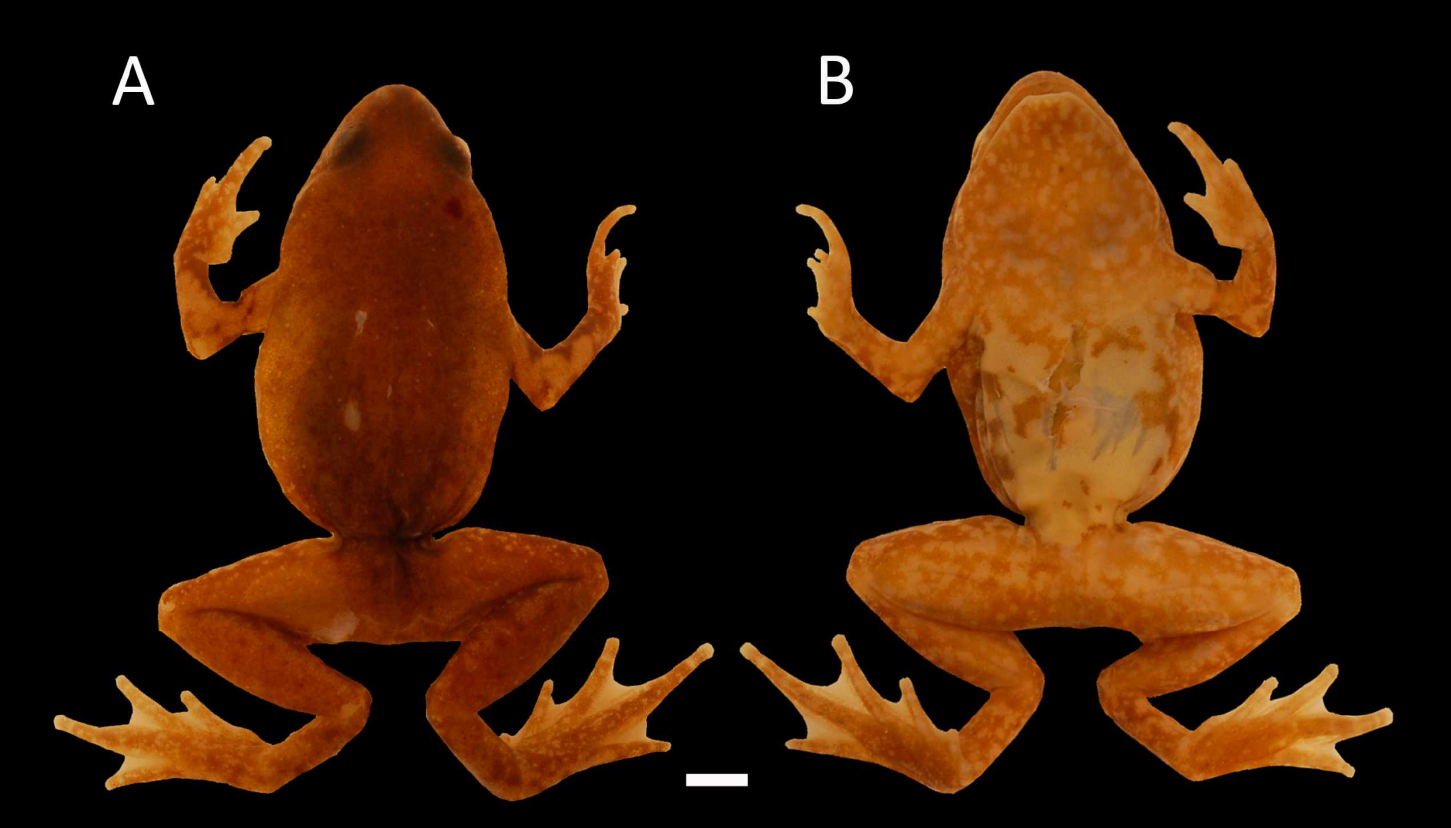
Holotype of *Chiasmocleis altomontana*. Holotype of *Chiasmocleis altomontana* sp. nov. MZUSP 133644, male; (A) Dorsal and (B) ventral views.

***Chiasmocleis altomontana*** **sp. nov.** (Fig. 8)

**Holotype.** MZUSP 133644 (Field Number 474), adult male, collected at Estação Ecológica de Bananal, Municipality of Bananal, State of São Paulo, Brazil (22°48′22″S; 44°22′08″W) on January 2004 by H Zaher.

**Paratypes.** MZUSP 131878, 133292, 135905, 131874, 131876, 131877, 131879, 133341, 133342, 133639, 133640, 133641, 133645, 133646, 133647, 135900, 133642 (Field Number 297, 1311, 1342, 128, 288, 296, 298, 1626, 1699, 419, 430, 456, 481, 489, 782, 1333, 458), females; MZUSP 131880, 133643 (Field Number 395, 461), males, all specimens collected in the same locality and collector of the holotype, between December, 2003 and January, 2004.

**Diagnosis.** A small-size species of *Chiasmocleis* (males *χ* = 17.2 mm, females *χ* = 20.2 mm; Table 2), diagnosed by the following characters: (1) body slender; (2) snout short, rounded in lateral and dorsal views; (3) ventral outline of the head, triangular; (4) first finger short in males; (5) fingers fringed, webbed in males; (6) toes fringed, webbed in males and females; (7) arms, legs, fingers, and toes with few small dermal spines in males; (8) tibia and thigh of about equal lenght; (9) combined thigh and tibia lengths abouy 90% of snout-vent length; (10) foot length abouy 51% of snout-vent length; (11) dorsal surface of the body with few small and uniformly distributed dermal spines in males and females; (12) vocal slits and sacs absent; (13) two smooth palatal ridges, first one pigmented; (14) male throat not infuscate; (15) venter light brown and cream, with scattered dark spots or markings, more intense on the throat.

**Table 2:** Measurements of *Chiasmocleis altomontana* sp. nov. Summary statistics of *Chiasmocleis altomontana* sp. nov.

Measurements	Male (*N* = 3)			Female (*N* = 17)		
	Range	$\overline{X}$	SD	Range	$\overline{X}$	SD
SVL	16.6–17.7	17.2	0.5	15.6–2.1	20.2	2.1
HL	3.4–4.0	3.7	0.2	3.3–4.5	4.0	0.3
HW	4.6–5.0	4.8	0.2	3.8–6.0	5.1	0.5
ED	1.2–1.3	1.3	0.0	1.1–1.7	1.4	0.1
IOD	2.4–3.0	2.7	0.3	2.4–3.1	2.9	0.1
IND	1.1–1.2	1.1	0.0	1.0–1.5	1.3	0.1
END	1.4–1.6	1.5	0.1	1.3–1.5	1.6	0.1
THL	7.8–7.9	7.8	0.0	6.3–9.3	8.3	1.0
TBL	7.4–8.0	7.7	0.3	6.2–9.3	8.3	0.9
FL	7.8–9.0	8.3	0.6	7.15–0.3	9.1	1.0
HDL	3.9–4.7	4.3	0.3	3.8–5.6	4.9	0.5
3FD	0.4–0.4	0.4	0.0	0.3–0.5	0.4	0.0
4TD	0.4–0.5	0.5	0.0	0.4–0.6	0.6	0.0
FAL	3.3–3.8	3.6	0.2	3.4–4.7	4.1	0.4

**Notes.**

N number of specimens X mean SD standard deviation

**Description of Holotype**. Body slender; head triangular, broader than long; snout short, snout tip rounded in dorsal and lateral views; nostrils located at the tip of snout, not protuberant, directed laterally; internostril distance smaller than eye–nostril distance and slightly smaller than eye diameter; canthus rostralis slightly defined; loreal region slightly convex; lips not flared; eyes small, only slightly protruding; upper eyelid well define; interorbital area flat; cranial crests absent; incomplete occipital fold; tympanum indistinct; upper jaw prognathus; mandible truncated with a trilobed anterior margin; tongue elongated, posteriorly unnotched; premaxillae, maxillae, and vomers teeth absent; choanae small, rounded, widely separated, positioned anterolaterally to eye; two smooth palatal ridges, first one pigmented and shorter than the second; vocal slit absent.

**Figure 9:**
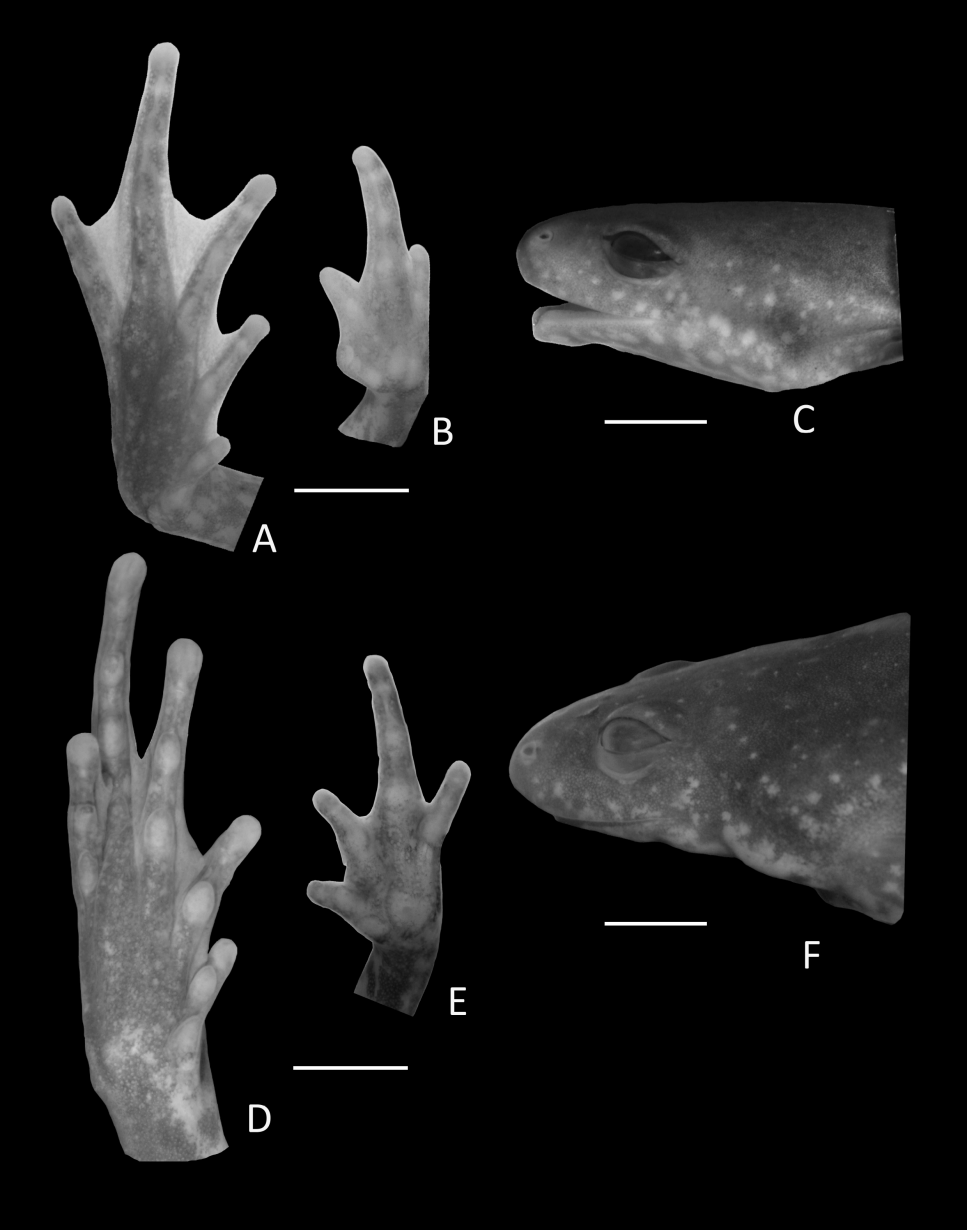
Details of Holotype *Chiasmocleis altomontana*. Ventral view of the foot, hand, and lateral profile of the head of *Chiasmocleis altomontana* sp. nov.; (A–C) Holotype MZUSP 133644, male, (D–E) Paratype MZUSP 133644, female.

Arms slender, lacking tubercles and crests on forearm. Hand strongly fringed and webbed (Fig. 9B); fingers lacking disks, with few dermal spines; fingers lengths I < II < IV < III; adpressed FI nearly reach the proximal subarticular tubercle of FII; adpressed FII slightly overlap the distal margin of the proximal subarticular tubercle on FIII; adpressed FIV reach the space between the proximal and the distal subarticular tubercle on FIII; thumb without nuptial asperities; subarticular tubercles well developed, rounded; supernumerary tubercles absent; thenar tubercle well developed, ovoid, at the base of finger I; palmar tubercle large divided into two parts inner one rounded and outer part elongated (Fig. 9B). Legs short moderately robust; knee and heel lacking tubercles; tibial and tarsal ridges absent. Foot of males (Fig. 9A) webbed, webbing passes the second subarticular tubercle of toes IV and V; toes fringed and lacking disks; toe lengths I < II < V < III < IV; adpressed TI does not reach the proximal subarticular tubercle on TII; adpressed TV reaches the distal margin of the middle subarticular tubercle on TIV; toes with few small lateral dermal spines; subarticular tubercles well developed and elongated; supernumerary tubercles absent; an oval inner metatarsal tubercle, no outer metatarsal tubercle (Fig. 9A).

Skin smooth above and beneath; dorsal surfaces of body and limbs with few small, uniformly distributed dermal spines. Anal opening not modified, lacking para-anal tubercles and glands around the cloaca.

**Figure 10:**
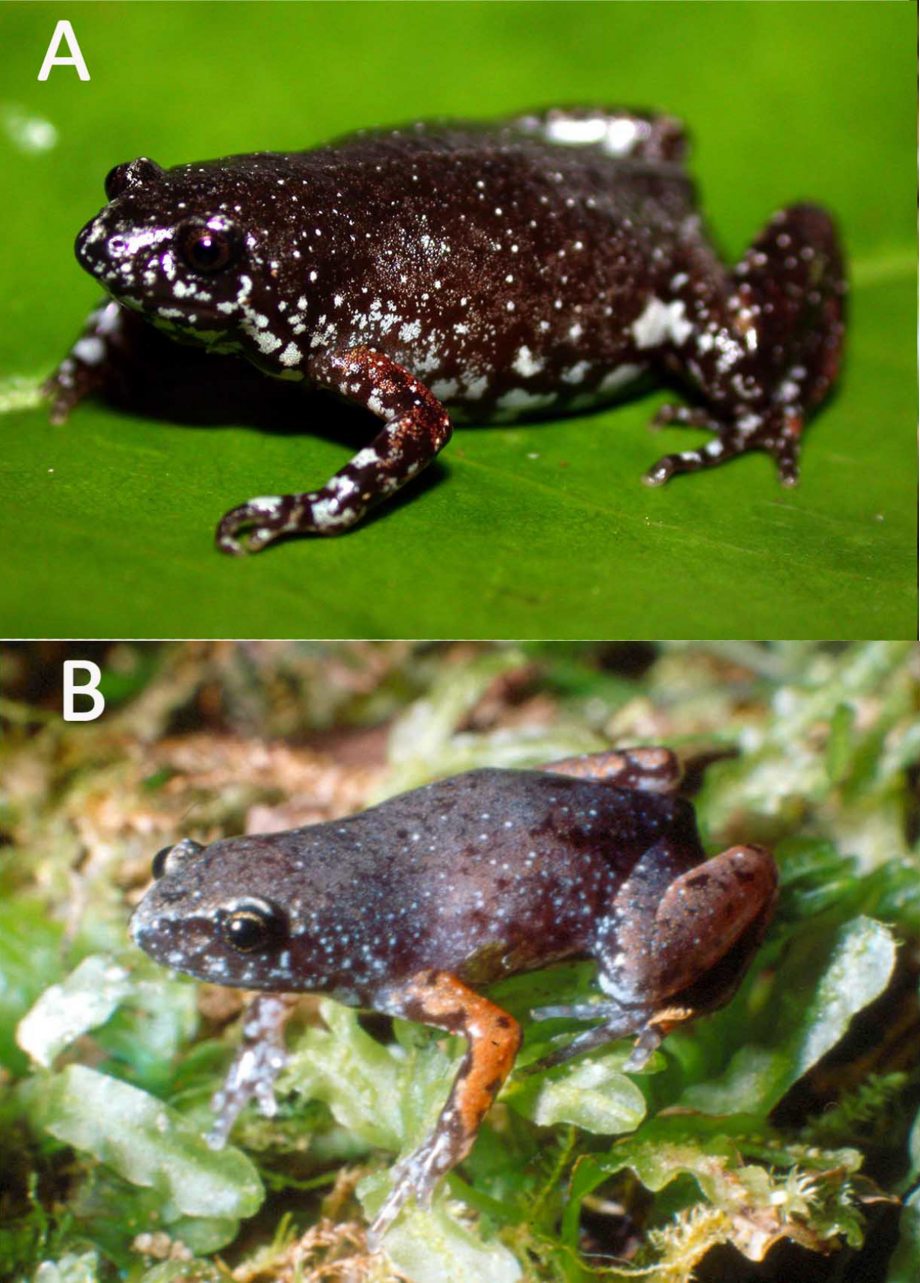
Live specimens. Live specimen of *Chiasmocleis altomontana* sp. nov.: (A) female adult; (B) juvenile; photo courtesy of PH Bernardo.

**Coloration in preservative.** Dorsum uniformly dark brown flanks light brown; venter cream with few scattered dark brown blotches dark blotches more common on the edges of the belly; throat pale brown with small cream spots and markikng; flanks marble light brown and cream; dorsal surface of arms and hands dark brown with large cream blotches; palms of hands marbled brown and pale cream; legs marbled dark brown and cream with few and scattered small cream spots; foot dorsally dark brown. Ventral surface of legs (i.e., thigh, tibia, and tarsus) pale brown with cream blotches. A light middorsal line is almost indistinguishable, running from the cloaca to the middle of the back; femoral line present (Fig. 8B). We did not examine coloration of live specimens (photo of alive specimen courtesy of PH Bernardo, Fig. 10)

**Measurements of Holotype.** SVL 17.7; HL 3.4; HW 5.0; ED 1.2; IOD 2.6; IND 1.1; END 1.5; THL 7.9; TBL 8.0; FL 9.0, HDL 4.7; FAL 3.6; 3FD 0.4; 4TD 0.54.

**Variation in the type series.** A summary of measurements is provided in Table 2. The types series overall agrees with coloration of the holotype, except for the cream blotches found on the dorsal surface arms and legs whih can be absent or more evident in some specimens. In three specimens the belly pattern is roughly marble in dark brown and pale cream. The throat can varied from pale brown with small cream blotches as that of the holotype to the described belly pattern of the holotype (i.e., pale cream, with scattered dark spots or stains). A dorsal midline is present in 65% of the specimens whereas a femoral line is present in 85% of the specimens. Only two specimens (a male and a female) show a transversal ventral line extending between the arms. Type series specimens have dermal spines on body dorsum, but they are absent on the posterior surface of the thigh, arms, legs, and hands in some females. Females have less developed webbing between the toes and lack webbing between the fingers (Figs. 9D–9F). Also, fingers and toes of females are less fringed distally. The length of FI when adpressed to the FII reaches the base of the proximal subarticular tubercle in FII; adpressed FIV reaches the base of the distal subarticular tubercle in FIII; adpressed TI reaches the proximal subarticular tubercle in TII in 75% of the females; adpressed TV touch the distal margin of the middle subarticular tubercle in TIV in 25% of the females. The combined mean thigh and tibia lengths represents approximately 83% of mean snout-vent length in males and 90% in females; foot length is about 48.3% of snout-vent length in males and 45.5% of females.

**Comparisons with other species**. The following species occur close to the distribution of *Chiasmcoleis altomontana* sp. nov., *C. atlantica*, *C. lacrimae*, *C. leucosticta*, and *C. mantiqueira*. The new species can be easily distinguished from three of those species by the lack of vocal slits on males (vocal slits present in *C. atlantica*, *C. lacrimae*, and *C.  leucosticta*); males and females with distinct webbed feet (feet not webbed in *C. atlantica*, *C. lacrimae*, Cruz, Caramaschi & Izecksohn, 1997 only basally webbed in *C. leucosticta*; Cruz, Caramaschi & Izecksohn, 1997); belly pattern cream with few scattered dark brown blotches (belly pattern: roughly marble in dark brown and pale cream in *C. atlantica* and *C. leucosticta*, pale cream without dark spots or blotches in *C. lacrimae,* Cruz, Caramaschi & Izecksohn, 1997); throat is not infuscated (infuscated in: *C. atlantica*, *C. lacrimae*, and *C.  leucosticta*, Cruz, Caramaschi & Izecksohn, 1997); dermal spines absent from body ventral surface of males (ventral surface of the belly and shin with dermal spines in *C. atlantica* and *C.  lacrimae*). The new species is morphologically similar to *C. mantiqueira* but it differ from this species by: less membrane among the fingers (membrane is more extensive in *C. mantiqueira*), ventral outline of the head is triangular (i.e., from the arms to the tip of the snout; rounded in *C. mantiqueira*) dermal spines on males and females are less abundant (spines abundant in *C. mantiqueira*) and first palatal grove is pigmented (not pigmented in *C. mantiqueira*, Cruz, Feio & Cassini, 2007). Furthermore, *C. altomontana* can be recognized by having a similar size (male SVL *χ* = 17.2 mm; female SVL *χ* = 20.2 mm) to *C. lacrimae* (male SVL *χ* = 17.1 mm; female SVL *χ* = 20.2 mm, Cruz, Caramaschi & Izecksohn, 1997 ), being larger than *C. mantiqueira* (male SVL *χ* = 16.5 mm; female SVL *χ* = 21.5 mm, Cruz, Feio & Cassini, 2007), and smaller than *C. atlantica* (male SVL *χ* = 23.1 mm, female SVL *χ* = 30.6 mm, Cruz, Caramaschi & Izecksohn, 1997) and *C. leucosticta* (male SVL *χ* = 20.0 mm; female SVL *χ* = 23.6 mm, Cruz, Caramaschi & Izecksohn, 1997).

*Chiasmocleis altomontana* sp. nov. can be distinguished from other Atlantic Forest species by: the absence of vocal slits in males (vocal slits present in *C. alagoana*, *C. capixaba*, *C. crucis*, *C. cordeiroi*, *C. gnoma*, *C. migueli*, *C. quilombola*, *C. sapiranga*, *C. schubarti*, and *C. veracruz*, Cruz, Caramaschi & Izecksohn, 1997; Cruz, Caramaschi & Freire, 1999; Caramaschi & Pimenta, 2003; Canedo, Dixo & Pombal, 2004; Cruz, Caramaschi & Napoli, 2007; Tonini, Forlani & de Sá, 2014); foot webbing present in males (foot webbing absent in *C. alagoana*, *C. gnoma*, and *C. veracruz*, Cruz, Caramaschi & Freire, 1999; Canedo, Dixo & Pombal, 2004); foot webbing that overlap the first and second subarticular tubercles between toes III and V (foot webbing does not overlap the first and second subarticular tubercles between toes III and V in *C. capixaba*, *C. crucis*, *C. migueli*, Cruz, Caramaschi & Izecksohn, 1997; present study, and *C. quilombola*, Tonini, Forlani & de Sá, 2014); female feet webbed (females feet not webbed in *C. alagoana*, *C. capixaba*, *C. gnoma*, *C. migueli*, *C. schubarti*, *C. quilombola*, and *C. veracruz*); male fingers webbed (fingers not webbed in *C. alagoana*, *C. capixaba, C*. *crucis, C*. *cordeiroi, C*. *gnoma, C*. *migueli, C*.* quilombola, C*. *sapiranga, C*. *schubarti*, and *C. veracruz*, Cruz, Caramaschi & Izecksohn, 1997; Cruz, Caramaschi & Freire, 1999; Caramaschi & Pimenta, 2003; Canedo, Dixo & Pombal, 2004; Cruz, Caramaschi & Napoli, 2007; Tonini, Forlani & de Sá, 2014); belly pattern cream with few scattered dark brown blotches (belly pattern: roughly marbled with dark brown and pale cream in *C. alagoana, C*. *crucis, C*. *cordeiroi, C*. *sapiranga,* and *C. schubarti*, Cruz, Caramaschi & Freire, 1999; Caramaschi & Pimenta, 2003; Cruz, Caramaschi & Napoli, 2007; brown with large cream blotches in *C. gnoma*, Canedo, Dixo & Pombal, 2004; pale cream without dark spots or blotches in *C. capixaba, C*. * migueli*, *C. quilombola*, and *C. veracruz*, Cruz, Caramaschi & Izecksohn, 1997; Tonini, Forlani & de Sá, 2014); throat not infuscated (throat infuscated in *C. alagoana*, *C. capixaba*, *C. crucis*, *C. cordeiroi*, *C. gnoma*, *C. migueli*, *C. sapiranga*, *C. schubarti*, *C. veracruz*, and *C. quilombola*, Cruz, Caramaschi & Izecksohn, 1997; Cruz, Caramaschi & Freire, 1999; Caramaschi & Pimenta, 2003; Canedo, Dixo & Pombal, 2004; Cruz, Caramaschi & Napoli, 2007; Tonini, Forlani & de Sá, 2014). Furthermore, *Chiasmocleis altomontana* sp. nov. also can be recognized by its smaller size (male SVL *χ* = 17.2 mm; female SVL *χ* = 20.2 mm) relative to *C. alagoana* (male SVL *χ* = 22.7–23.4 mm; female SVL *χ* = 26.8 mm, Cruz, Caramaschi & Freire, 1999), *C. cordeiroi* (male SVL *χ* = 21.5 mm; female SVL *χ* = 25.9 mm), *C. crucis* (male SVL *χ* = 20.6 mm), *C. migueli* (male *χ* = 20.0 mm; female SVL *χ* = 18.1 mm), *C. sapiranga* (male SVL *χ* = 19.9 mm; female SVL *χ* = 23.5, Cruz, Caramaschi & Napoli, 2007), *C. schubarti* (male SVL *χ* =23.5 mm; female SVL *χ* = 28.7, Cruz, Caramaschi & Izecksohn, 1997. Moreover, the new species is larger than *C. capixaba* (male SVL *χ* = 15.6 mm, Cruz, Caramaschi & Izecksohn, 1997), *C. gnoma* (male SVL *χ* = 13.9 mm; female SVL *χ* = 15.9 mm, Canedo, Dixo & Pombal, 2004), *C. quilombola* (male SVL *χ* = 14.4 mm; female SVL *χ* = 17.1 mm, Tonini, Forlani & de Sá, 2014), and *C. veracruz* sp. nov. (male SVL *χ* = 15.3 mm, female SVL *χ* = 16.7 mm).

**Distribution.** *Chiasmocleis altomontana* sp. nov. is only known from the type locality, Estação Ecológica do Bananal, Municipality of Bananal, State of São Paulo, Brazil (Fig. 4). *Chiasmocleis altomontana* is the second species in the genus reported to occur exclusively at high altitude (*C. mantiqueira* occurs exclusively in high elevation mountains as well).

**Etymology** The specific name is a combination derived from the Latin words *altus* meaning high and *montani* meaning mountain that characterizes the high altitudinal mountain distribution up to 1,000 m of the new species.

**Conservation.** *Chiasmocleis altomontana* sp. nov. was found primarily inside forest fragment in State protected area. Although the current range of *Chiasmocleis altomontana* sp. nov. is restricted to the type locality, the species is not threatened locally; it was reported to occur in good abundance at the type locality (Zaher, Aguiar & Pombal Jr, 2005).

### Osteology of the new species

**Table:** 

**Figure 11:**
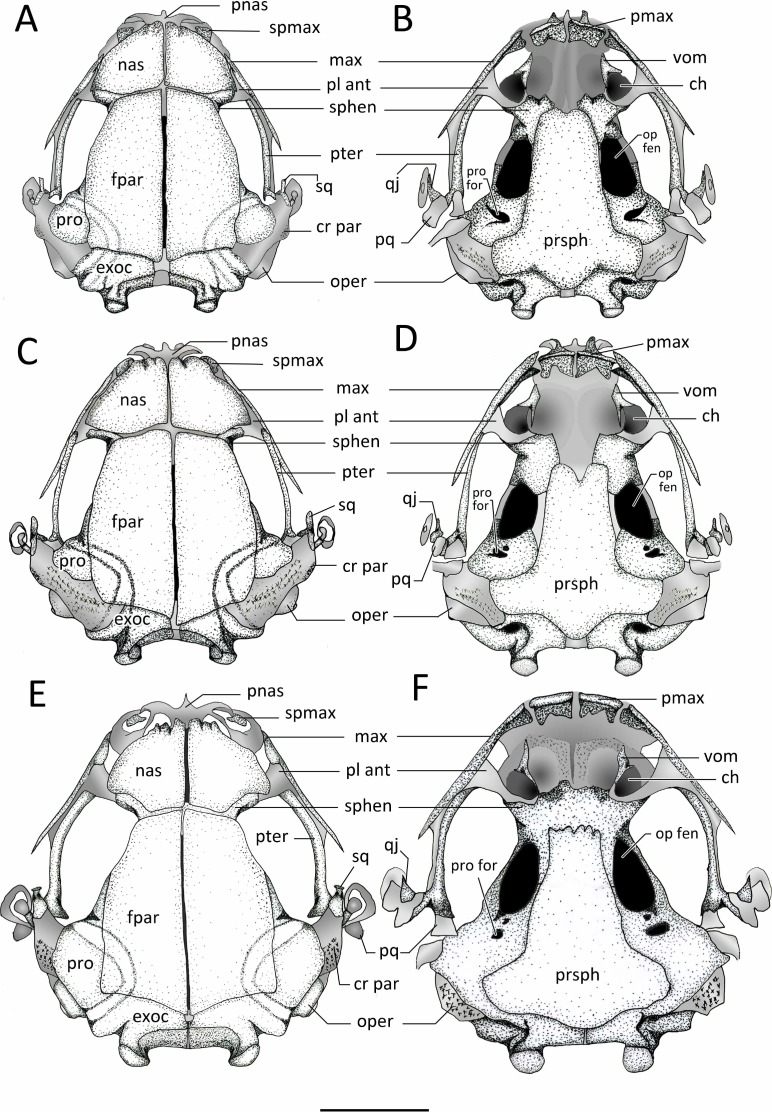
Dorsal and ventral view of skulls. Dorsal and ventral views of the skull of: (A–B) *Chiasmocleis veracruz* sp. nov. (UFBA 3578), (C–D) *Chiasmocleis migueli* sp. nov. (MZUSP 114584), (E–F) *Chiasmocleis altomontana* sp. nov. (MZUSP 131875). Abbreviations: ch, choana; cr par, crista parotica; exo, exocciptal; fpar, frontoparietal; qj, quadratojugal; max, maxilla; nas, nasal; oper, operculum; op fen, optic fenestra; pl ant, planum atorbitale; pmax, pre maxilla; pnas proc, prenasal process; pq, palatoquadrate; pro, prootic; pro for, prootic foramen; pter, pterygoid; prsph, parasphenoid; sq, squamosal; sphe, sphenethimoid; spmax, septomaxillaae; vom, vomer. Gray represents cartilage; stippled gray is mineralized cartilage. Bar, 2 mm.

**Figure 12:**
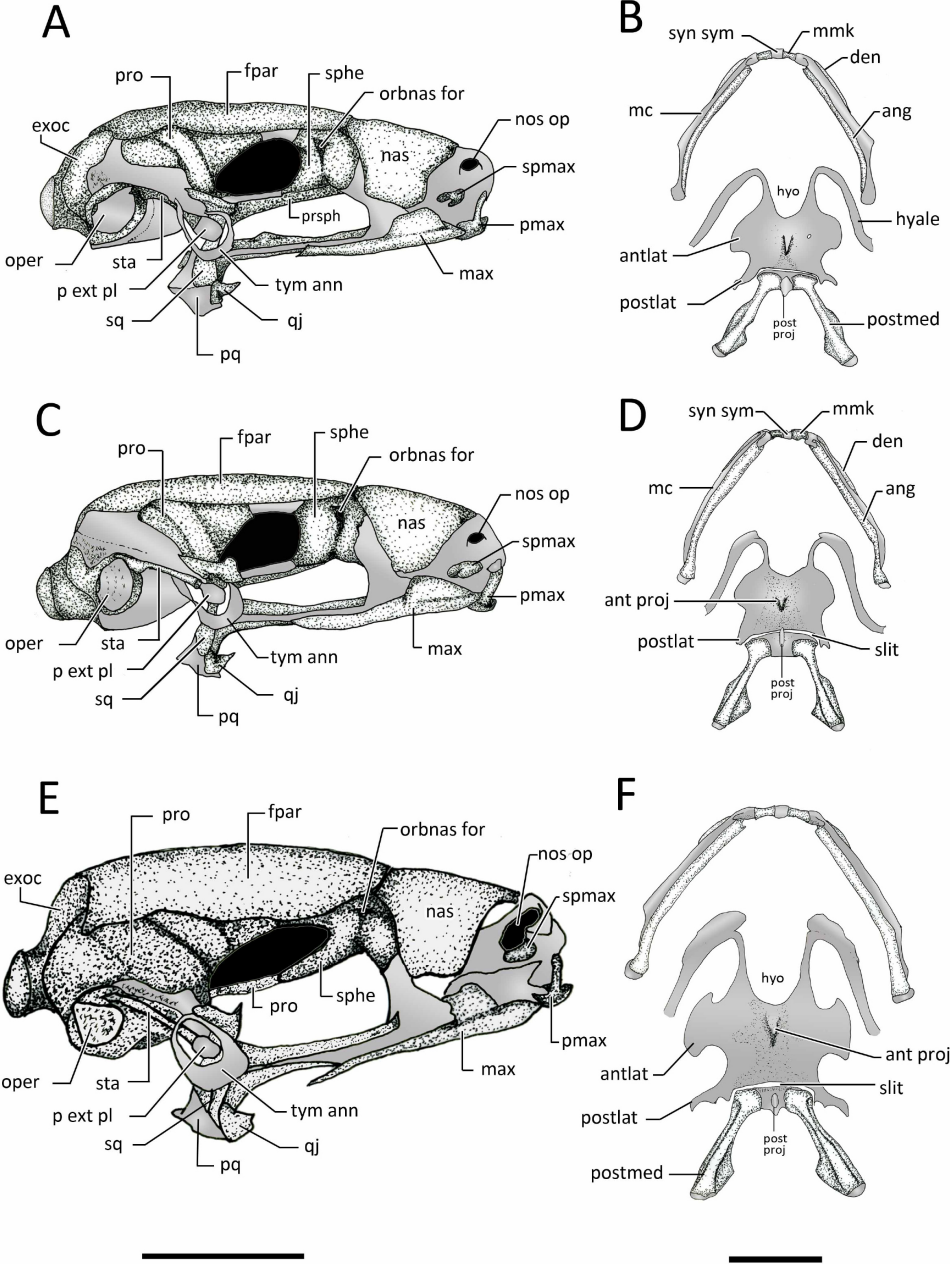
Lateral view skull and ventral view of hyod apparatues. Lateral views of the skull and ventral view of mandible hyoid apparatus of: (A–B) *Chiasmocleis veracruz* sp. nov. (UFBA 3578), (C–D) *Chiasmocleis migueli* sp. nov. (MZUSP 114584), and (E–F) *Chiasmocleis altomontana* sp. nov. (MZUSP 131875). Abbreviations: ang, angulosplenial; antlat, anterolateral process; ant proj, anterior projection; den, dentary; exo, exocciptal; fpar, frontoparietal; hyo, hyoglossal foramen; max, maxilla; mc, Meckel’s cartilage; mmk, mentomeckelian bone; nas, nasal;nos op, nostril opening; orbnas for, orbitonasal foramen; oper, operculum; op fen, optic fenestra; p ext pl, pars external plectri; pmax, pre maxilla; postlat, posterolateral process; postmed, posteromedial process; post proj, posterior projection; pq, palatoquadrate; pro, prootic; qj, quadratojugal; sq, squamosal; sphe, sphenethmoid; spmax, septomaxillae; sta, stapes; syn syn, syndesmotic symphysis; tyn ann, tympanic annulus. Gray represents cartilage; stippled gray is mineralized cartilage. Bar, 2 mm.

**Cranial Osteology** (Figs. 11 and 12).

The skulls of *Chiasmocleis veracruz* and *C. migueli* are well ossified, with cartilaginous areas that show some ossification. The skull of *C. altomontana* is the most ossified of the three species. The skulls are widest at the level of the jaw articulation in the three new species. However, the skull is overall ovoid, dorsoventrally flat, and slightly longer than wide in *Chiasmocleis veracruz*; whereas the other two new species have the opposite conditions. The jaw articulation lies far anterior to the otic capsule. The arch of the planum antorbitale is directed anterolaterally forming the posterolateral walls of the nasal capsules and the anterior wall of the orbits. The posterolateral walls of the auditory capsules and the crista parotica are mostly cartilaginous and poorly ossified. The olfactory capsule in *C. veracruz* is cartilaginous whereas in *C. altomontana* is mineralized and heavily mineralized in *C. migueli.*

**Endocranium***. Sphenethmoids*. The paired sphenethmoids are well developed and form the anterolateral wall of the neurocranium and the anterior margin of the optic fenestra. Dorsally, the sphenethmoids are visible between the nasals and the frontoparietals on the anterior margin of the optic fenestra; they extend anteriorly but do not reach the posterior border of the choanae. They are fused into a single element in *C. altomontana* (Fig. 11). Moreover, the sphenehmoids extend posteriorly over the anterior third of the optic fenestra and contribute to form its ventral and dorsal edges (Fig. 12); the sphenethmoid of *C. altomontana* is more extensive and contacts with the prootics, encapsulating the optic fenestra in bone. The anterior margins of the sphenethmoids surpass the posterior edge of the choana but do not reach the level of the medial ramus of the vomer.

*Prootics and exoccipitals*. The exoccipital and prootic are synostotically fused and have a cartilaginous connection to the crista parotica. The crista parotica is not ossified but slightly calcified on its lateral edge. Epiotic eminnences are visible. The prootics form the posterolateral walls of the neurocranium, the posterior margins of the optic foramina, and the anterior and ventrolateral areas of the otic capsules. Ventrally, the medial and posterior margins of the prootics are slightly overlapped by the parasphenoid. Dorsally, their anteromedial margins are overlapped by the frontoparietals. The exoccipitals ossified on the posteromedial wall of the neurocranium, contributing to form the posterior part of the otic capsules, the margins of the foramen magnum, and the occipital condyles; the occipital condyles bear round articular surfaces. Dorsally, the anteromedial margins of the exoccipitals are overlapped by the frontoparietals and ventrally by the alae and posteromedial process of the parasphenoid. The prootics and exoccipitals are separated by cartilage in *C. veracruz* and *C. migueli*, whereas in *C. altomontana* this cartilaginous area is fully ossified (Fig. 11). Dorsally, the exoccipitals are fused medially in *C. altomontana*, whereas in *C. migueli* and *C. veracruz* are separated by cartilage.

*Plectral apparatus.* The plectral apparatus is found ventrolaterally to the crista parotica. The collumela (*pars media plectri*) is a long and cylindrical bone with the distal end flattened and expanded to form a cartilaginous, ventral, *pars external plectri* (Fig. 12). The *pars externa plectri* is surrounded by a wide ring of flat cartilage that contacts with the cartilaginous crista parotica. The proximal end of the collumela expands and flattens to contact with the operculum. The operculum is well developed, mostly cartilaginous, and occludes the fenestra ovalis. The operculum is heavily mineralized in *C. altomontana*.

**Exocranium***. Nasal.* The paired nasals are expanded and well-ossified bones, covering almost completely the olfactory capsules; they extend posteriorly from the level of the alary cartilages and the anterior ends of the septomaxillae to reach the sphenethmoids, they do not contact with the frontoparietals. Medially, the nasals are narrowly separated, they do not articulate with the frontoparietals or maxillae, and posteriorly overlap the sphenethmoids. The nasals curve ventrolaterally but they do not reach the maxillae. The anterior margin of the nasal is slightly concave with irregular ossification. The cartilaginous septum nasi is visible between the nasals. The nasals are overall triangular and similar in the three new species, but in *C. altomontana* they have narrower posterior margins so they have four distinct edges. In the three new species, the nasals have descending maxillary processes, which do not contact with the preorbital process of the maxillae; the descending maxillary process is more pronounced in *C. altomontana.*

*Frontoparietals.* The frontoparietals are broad paired bones, overall rectangular with rounded lateral edges, and slanted anterior edges. They are well ossified, lack a supraorbital flange, are narrowly separated medially along their length, and almost completely roofing the frontoparietal fontanelle. The anterior margins do not articulate with the nasals but overlap the sphenethmoids. Posteriorly the frontoparietals overlap the area of contact between the prootic and exoccipitals.

*Parasphenoid.* The parasphenoid lacks ornamentation. The cultriform process is wide, overall rectangular shaped, and overlaps the posterior margin of the sphenethmoids. The parasphenoid alae are short and wide; the width of the cultriform process gently decreases towards its anterior end. The alae are oriented posterolaterally beneath the otic capsules and they are widely separated from the medial ramus of the pterygoid. The posteromedial process of the parasphenoid is distinct, rounded, and underlies the cartilaginous margin of the foramen magnum between the ossified exoccipitals. The anterior margin of the cultriform process bears an irregular margin in *C. altomontana*, whereas it is slighlty notched in *C. veracruz* and distictly V-shaped notched in *C. migueli*. Additionally, the anterior edge of the alae is slightly angled posteriorly in *C. veracruz*, horizonal in *C. migueli*, and distinctively angled posteriorly in *C. altomontana*. Moreover the parasphenoid distinctly overlaps the sphenethmoids, prootics, and exoccipitals in *C. altomontana* and *C. veracruz* but only slightly in *C. migueli*.

*Vomer.* The paired anterior vomers are small and lack articulation with adjacent elements; posterior vomer are absent. Ventrally, the vomers are slender and triangular shaped; the posterior and medial (i.e., prechoanal) processes form the anterolateral margin of the choanas; a smaller dorsal process is also present. Vomerine odontophores, teeth, and odontoids are absent.

*Premaxillae*. The edentate premaxillae are narrowly separated medially and do not contact with the maxilla laterally. The alary processes of the premaxillae are well developed, slightly inclined anteriorly and ending in a curved cartilaginous tip. The pars palatina are broad and have well-developed medial and lateral processes.

*Septomaxillae.* The septomaxillae are relatively large and deeply curved bones lying within the nasal capsule. They are clearly visible in dorsal and lateral views, located anterolaterally to the nasals and between the nasal and the *pars facialis* of the maxilla.

*Maxillae*. The maxillae lack pre and postorbital processes and teeth. Anteriorly, the maxillae do not contact but slightly overlap the premaxillae at the level of the facial process. Posteriorly, the maxillae extends to overlap the pterygoids for about half of their length, and do not contact with the quadratojugals. The preorbital process of the maxilla is broadest at its anterior third, reaching its maximal depth posteriorly to the septomaxilla. The preorbital process of the maxilla extend dorsal towards, but does not contact, the descending maxillary process of the nasal and decreases posteriorly. The pars palatina is poorly developed, bearing a low triangular process at the anterior end. The preorbital process of the maxilla is overall square, broad, and low in *C. veracruz*, triangular in *C. migueli*, and square and narrow in *C. altomontana*. The anterior edge of the pars facialis of the maxilla overlaps the premaxilla more markedly in *C. migueli*, being visible in lateral and frontal views (Fig. 12).

*Quadratojugals.* The quadratojugals are very short, triangular and curved towards the maxillae. The anterior tips are sharp and the posterior ends contact with the ventrolateral tip of the pars articularis of the cartilaginous palatoquadrate.

*Mandible*. The mandibles lack ridges or odontoids. The dentaries are a thin and elongated bones overlaping the anterior half of the anterolateral outer surfaces of Meckel’s cartilage, whereas the angulosplenials overlap most of the inner and ventral surface of Meckel’s cartilage. Anteriorly, the dentaries are wider and dorsally fused to the mentomeckelian bones located on either side of the cartilaginous mandibular symphysis. The lateral end of each mentomeckelian is associated with a poorly developed cartilaginous mentomeckelian diverticulum which lies slightly medial and adjacent to the anterior tip of the angulosplenial.

**Suspensorium***. Pterygoid.* The medial ramus of the pterygoid is short and does not contact the otic capsule, parasphenoid, or branincase. The anterior ramus is long, arched, and overlaps the maxillary cartilage and reaches the planum anteorbitalis. The posterior ramus is shorter and wider (distinctly wider in *C. altomontana*) than the anterior ramus and overlaps the lateral surface of the palatoquadrate.

*Squamosal.* The squamosal is overall “T”-shaped, bearing a long ventral ramus and short otic and zygomatic rami. The otic ramus is about three times longer than the zygomatic ramus and articulates with the cartilaginous anterolateral margin of the crista parotica. The ventral ramus overlies the palatoquadrate laterally and its ventral tip contact the quadratojugal. The ventral ramus is slightly posterior and forms a 50°–80° angle with the maxilla. The zygomatic ramus of *C. altomontana* bears a dorsal process (Fig. 12) and it is three times shorter than the otic ramus.

*Palatoquadrate.* The palatoquadrate is cartilaginous; its external surface is covered by the ventral ramus of the squamosal whereas its inner surface is overlaped by the posterior ramus of the pterygoid.

**Hyobranchial skeleton.** *Hyoid* (Fig. 12). The hyoglossal sinus is “U”shaped with no traces of membrane. The hyoid is cartilaginous and poorly mineralized posteromedially; it bears a discrete medial process. The anterolateral processes of the hyoid plate are wide lateral expansions whereas the posterolateral processes are slender and posteriorly curved. The hyale are homogenous in width. A slit is found between the posterior margin of the hyoid plate and the anterior margins of the posteromedial processes. The posteromedial bones are connected by cartilage; this cartilage is slightly mineralized on its anterior margin. The posteromedial processes are long, slender, and expanded distally with lateral flanges. The hyoid plate of *C. altomontana* and *C. migueli* are strongly mineralized from its anteromedial to posteromedial margins (Fig. 12).

**Figure 13:**
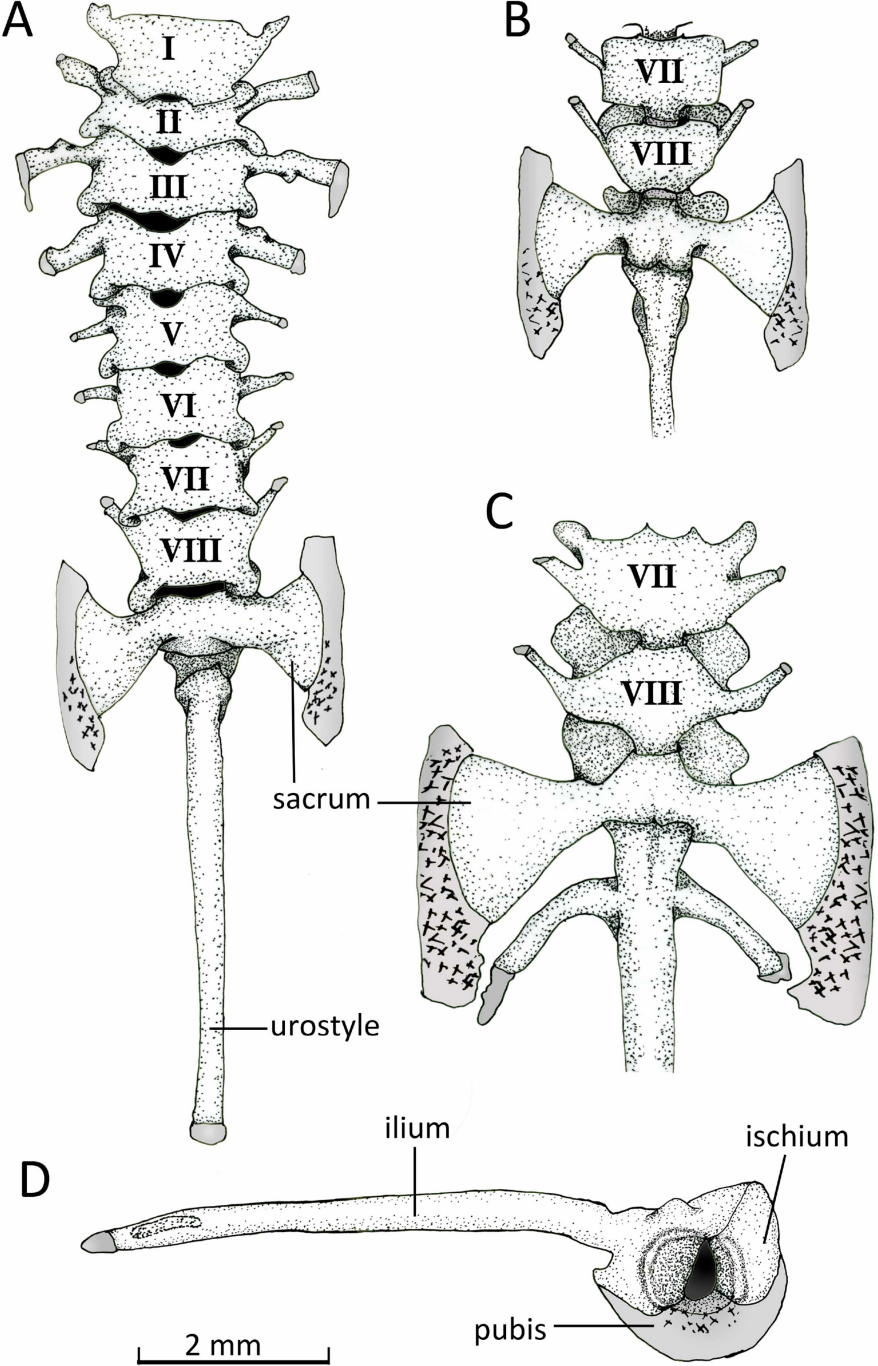
Vertebral column. Axial column with numbered presacral vertebrae of *Chiasmocleis veracruz* sp. nov. (UFBA 3578) in (A) dorsal and (B) ventral view of the last vertebrae. (C) Ventral view of the last vertebrae of *Chiasmocleis altomontana* sp. nov. (MZUSP 131875). (D) Lateral view of the pelvic girdle of *Chiasmocleis veracruz* sp. nov. (UFBA 3578). Gray represents cartilage; stippled gray is mineralized cartilage. Bar, 2 mm.

### Postcranial osteology

**Axial Skeleton.** *Vertebral column* (Fig. 13). The vertebral column consists of seven procoelous presacral vertebrae (I–VII), whereas presacral VIII is diplasiocoelous (Fig. 13). The cervical cotyles are widely separated. The presacral are nonimbricate, without neural spines, and approximately of equal size, being wider than long (except vertebra II). Witdh of presacral and sacral vertebrae, including transverse processes, are: III > sacrum > II > IV > V = VI > VII = VIII > I. The transverse processes of presacrals II–IV are expanded, whereas those of presacrals V–VII are thin (about half the width of the anterior ones). The transverse processes of presacral III have a medial anterodorsal projection. The transverse processes of presacrals III, V, and VI are oriented nearly perpendicular whereas those of presacral II are oriented slightly anteriorly, presacral IV slightly posteriorly, and presacrals VII–VIII are distinctly directed anteriorly. Sacral diapophyses are perpendicular to the midline, unequally expanded, and with their posterior halves being more than twice the width of the anterior halves. A mineralized cartilage is found on the lateral margins of the sacral diapophyses which articulates with the anterior tip of the ilial shaft. The urostyle is rounded and smooth, bearing a flat small lateral projection anteriorly. The cartilaginous edges of the sacral diapophyses of *C*. *altomontana* extend posteriorly to the ossified transverse processes to a greater extent and show more mineralization than those of *C. migueli* and *C. veracruz*. Also, *C. altomontana* has transverse processes on the urostyle immediately behind the sacral vertebra; we consider this an anomaly (Fig. 13C).

**Figure 14:**
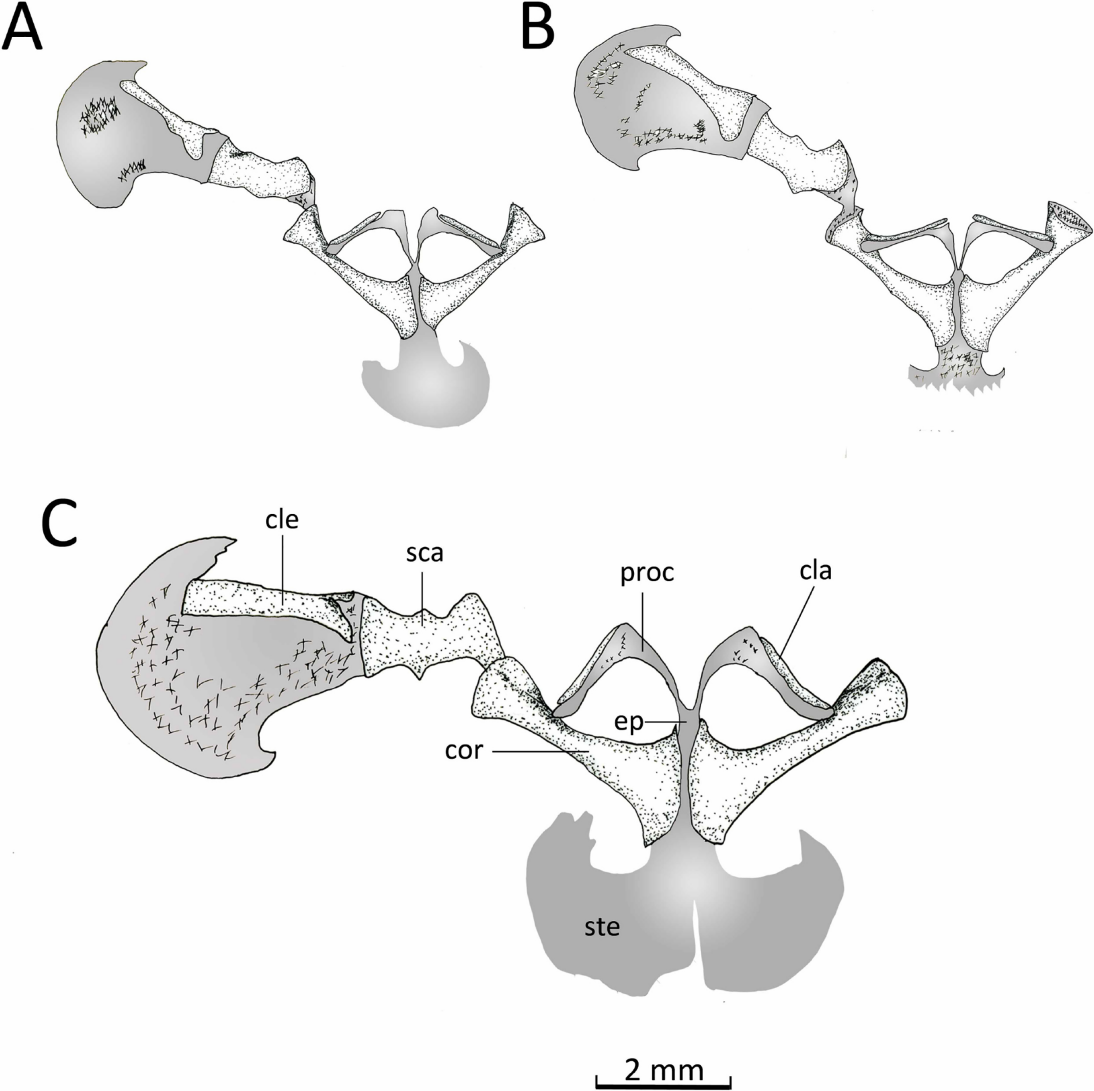
Ventral view pectoral girdle. Ventral views of the pectoral girdles of: (A) *Chiasmocleis veracruz* sp. nov. (UFBA 3578), (B) *Chiasmocleis migueli* sp. nov. (MZUSP 114584), and (C) *Chiasmocleis altomontana* sp. nov. (MZUSP 131875). Abbreviations: cla, clavicle; cle, cleithrum; cor, coracoid; ep, epicoracoid; proc, procoracoid; sca, scapula; ste, sternum. Gray represents cartilage; stippled gray is mineralized cartilage. Bar, 2 mm.

**Appendicular skeleton.** *Pectoral girdle* (Fig. 14). The firmisternal pectoral girdle bears a reduced procoracoid, a coracoid, clavicles, and fused epicoracoids. A wide and flat plate sternum is synchondrostically fused with the epicoracoid cartilage. The epicoracoids occupy the space between the coracoids and are continuous with the procoracoids. The paired procoracoid cartilages are moderated developed, extending continuously and arching laterally from the epicoracoids to the distal portion of the coracoids. The procoracoids are thin at the epicoracoid end and wider towards the clavicles. The clavicles are short, straight, and slender bones. The clavicles are supported by the procoracoid cartilages throughout their lengths. The coracoids are long and robust bones, narrowly separated medially by the epicoracoid cartilage, and connecting with the scapulae laterally at the glenoid fossa. In *C. altomontana* the coracoid contact directly with the the scapula, wheres in *C. migueli* and *C. veracruz* they connect through cartilage. At the sternum end, the width of the coracoid is about twice the width at the glenoid fossa and three times at its midshaft. The scapula is longer than wide, but shorter and more robust than the coracoid. The glenoid fossa is indistinct from the fused pars acromialis and glenoidalis. The cleithrum is thin and lies at the anterior margin of the slightly mineralized suprascapular cartilage, extending along most of its lenght. The proximal end of the cleithrum is expanded for about half its length. The suprascapula expands widely, being the distal margin almost three times its width at the glenoid fossa; distally it is distinctly “hooked.” The angle between the clavicle and the midline of the body is obtuse in *C. altomontana*, whereas *C. migueli* and *C. veracruz* have narrower angles (Fig. 14). The sternum of *C. altomontana* lacks mineralization whereas those of *C. migueli* and *C. veracruz* may have some mineralization. The scapulae of *C. altomontana* and *C. migueli* have a distinct medial process; smaller or absent in *C. veracruz*. The cleithrum is most narrow in *C. veracruz*. Moreover, the suprascapula is heavily mineralized in *C. altomontana* and, as in *C. migueli*, it has both anterior and posterior hooks; it is less mineralized and has a single hook in *C. veracruz* (Figs. 14A, 14C).

*Pelvic girdle* (Fig. 14D). In dorsal view, the space between the ilial shafts is U-shaped, the preacetabular angle of the ilium is about 90°. The antero and posterodorsal areas of the acetabulum are formed by the ilium and ischium, whereas the ventral portion is formed by a cartilaginous pubis. The pubis is completely calcified in *C. altomontana* and mineralized in *C. migueli*. *Chiasmocleis altomontana* bears a small crest dorsal to the acetabulum.

**Figure 15:**
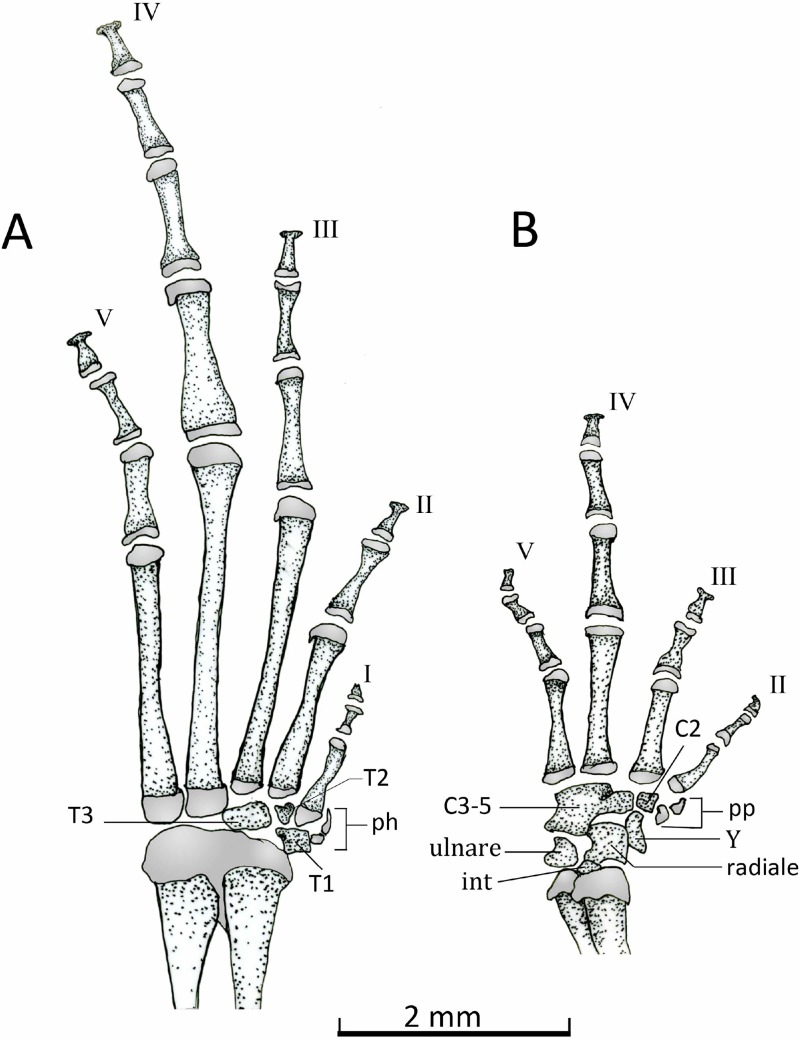
Dorsal view of feets and hands. Dorsal view of pes (A) and manus (B) of *Chiasmocleis veracruz* sp. nov. (UFBA 3578). Abbreviations: C2, distal carpal, C3–5, distal carpals, int, intermedium, ph, prehallux, pp, prepollex, T1–3, distal tarsals 1, 2, 3, Y, element Y. Gray represents cartilage. Black bar, 2 mm.

*Manus* (Fig. 15B). The phalangeal formula is 0-2-2-3-3; length of digits is IV > V > III > II (Fig. 15B). The distal ends of the terminal phalange are rounded and slightly expanded (knob shape), except on phalange II that is not expanded. Proximally the carpus consists of a large radiale, an ulnare, and a small and dorsally positioned intermedium. Carpal elements 3–5 are fused into a single element, which lies at the base of the metacarpals III–V. A single distal carpal 2 lies at the base of metacarpal II and lateral to metacarpal III. The prepollex is formed by a proximal bone and a small distal cartilaginous element. Element Y lies between the posterior margins of the prepollex and carpal 2 and anterior to the radiale. The prepollex of *Chiasmocleis migueli* remains cartilaginous, but the proximal element is heavily mineralized. Also in this species, the distal ends of the terminal phalanges are more expanded and not as rounded as in the other species.

*Pes* (Fig. 15A). The phalangeal formula is 2-2-3-4-3; digits length is IV > III > V > II > I. The distal ends of the terminal phalanges are slightly expanded. The tarsus bears a distal tarsal 1, 2, and 3 and a prehallux with two elements, being the distal one cartilaginous.

## Discussion

Incongruence between concatenation and species tree have been extensively report in the literature (Tonini et al., 2015) and it is still a topic of fervorous debate (Gatesy et al., 2016). Future analyses including more extensive taxa and gene sampling may converge in a single and robust phylogenetic hypothesis of *Chiasmocleis* species endemic of the Atlantic Forest.

The two phylogenetic analyses used (i.e., concatenation and the species tree) recovered well-supported clades of two new species: one as the sister taxon to *Chiasmocleis quilombola* and the other one as the sister taxon to *C. mantiqueira*. Genetic data has been useful to identify species that are morphologically very similar but represent separate evolving lineages, i.e., cryptic species (Rakotoarison et al., 2015; Scherz et al., 2016a; Scherz et al., 2016b; Streicher et al., 2012). The genetic distance in the 16S marker among *Chiasmocleis* species showed that the new species fall within the range of other recognized *Chiasmocleis* species. The lower values correspond to *C. capixaba*- *C. quilombola* (1.0), *C. quilombola*- *C. lacrimae*, *C. schubarti*- *C*. *crucis* and *C. altomontana*- *C. mantiqueira* (three pairs at 1.1). The genetic distances are comparable to those of other species described solely on morphology, e.g., *C. schubarti* and *C. crucis*. The higher values for the species pairs correspond to *C. mantiqueira*- *C. veracruz* (11.9), *C. mantiqueira*- *C. lacrimae* and *C. altomontana*- *C*. *veracruz* (11.1) (Table 3). The molecular phylogeny, along with the morphological characteristics that differentiate the species from other species occuring in the Atlantic Forest, support the recognition of the new taxa. Other life history data (e.g., larvae) and ecological and behavioral data (e.g., advertisement calls) may provide additional characteristics for the species.

**Table 3:** Genetic distances (p-uncorrected) in 16S marker among *Chiasmocleis* species used in the phylogenetic analysis (Fig. 1).

	ala	alt	cap	cor	cru	lac	leu	man	qui	sch	shu	ver
ala		1.6	1.3	0.5	0.8	1.2	1.6	1.6	1.2	0.7	1.7	1.3
alt	9	–	1.9	1.7	1.7	1.8	1.4	0.6	1.8	1.7	1.9	1.8
cap	5.7	10.6	–	1.3	1.3	0.5	1.6	1.8	0.5	1.3	1.8	0.8
cor	1.7	9.4	5.2	–	0.6	1.2	1.6	1.6	1.2	0.4	1.7	1.2
cru	2.9	10	5.2	2.2	–	1.2	1.6	1.7	1.2	0.5	1.7	1.2
lacr	5.4	10.4	1.3	4.9	4.9	–	1.6	1.7	0.4	1.2	1.7	0.7
leu	8.1	6.9	8.6	8.9	8.4	9	–	1.4	1.6	1.6	2.1	1.5
man	9.8	1.1	10.8	10.2	10.8	11.1	7.7	–	1.7	1.7	1.8	1.7
qui	5	10.1	1	4.5	4.6	1.1	8.7	10.9	–	1.2	1.8	0.6
sch	2.2	9.4	4.6	1.3	1.1	4.3	7.9	10.2	3.9	–	1.7	1.2
shu	13.9	13.3	13.5	14.1	14.3	13.8	14.2	13.8	13.6	13.9	–	1.7
ver	5.9	11.1	2.4	5.4	5.4	2.5	9.6	11.9	1.5	4.8	14.8	–

**Notes.**

ala alagoana alt altomontana cap capixaba cor cordeiroi cru crucis lac lacrimae leu leucosticta man mantiqueira qui quilombola sch schubarti shu shudikarensis ver veracruz

Lower diagonal values correspond to percentage genetic distances and upper diagonal values correspond to standard errors.

Characters that have been useful for species diagnosis of *Chiasmocleis* are: webbing between fingers and toes, belly pattern, distribution of dermal spines in males and females, and vocalizations (Cruz, Caramaschi & Izecksohn, 1997; Caramaschi & Pimenta, 2003; Canedo, Dixo & Pombal, 2004; Cruz, Caramaschi & Napoli, 2007). The distribution of the genus *Chiasmocleis* in the northern Atlantic Forest has been previously discussed (Van Sluys, 1998; Cruz, Caramaschi & Freire, 1999; Caramaschi & Pimenta, 2003). The discovery of *C. migueli* in the State of Sergipe provides a continuous distribution for the genus throughout the Atlantic Forest. Furthermore, it strengthens the need of additional sampling in that region (Caramaschi & Pimenta, 2003).

Fifteen *Chiasmocleis* species are endemic to the Atlantic Forest (Fig. 4). Biogeographically, the Atlantic Forest can be divided into Northern (NAF), Central (CAF), and Southern (SAF) areas (Silva et al., 2012). *Chiasmocleis alagoana* is the only *Chiasmocleis* endemic to the NAF. The CAF is the most diverse with nine species; seven of the species are endemics to the area: *C. cordeiroi*, *C. crucis*, *C*. *gnoma*, *C. migueli*, *C. sapiranga*, *C*. *quilombola*, and *C. veracruz*, whereas *C. capixaba* and *C. schubarti* also occur in SAF. Five species are endemic of the SAF: *C*. * altomontana*, *C. atlantica*, *C. lacrimae*, *C. leucosticta*, and *C. mantiqueira.*

*Chiasmocleis altomontana* and *C. mantiqueira* are the only high altitude (up to 1,000 m) Atlantic Forest species of the genus and they are sister taxa (Fig. 1). Consequently, it is not surprising that they are among the pairs of species with lowest genetic divergence value; although they comprise well-supported distinct clades and they are geographically isolated. Speciation of these taxa could have been the result of geographic isolation at mountains peaks (Lara & Patton, 2000; Clemente-Carvalho et al., 2009; Clemente-Carvalho et al., 2011). *Chiasmocleis altomontana* occupies the Serra do Mar mountain range south of the Paraiba do Sul river, whereas *C. mantiqueira* is found throughout the Serra da Mantiqueira mountain range north of the Paraiba do Sul river (northern end of São Paulo State to Minas Gerais State). Both species lack vocal sacs and vocal slits (*C. mantiqueira* in Santana et al., 2012, *C. altomontana* reported here) and females have well-developed feet membrane and dermal spines. The two species are sister taxa and these traits most likely were inherited from their common ancestor. Moreover, speciation of these taxa is likely to have ocurred in allopatry, i.e., isolated on mountain ranges of the Atlantic Forest as reported for other anurans and mammals (Pombal Jr & Haddad, 1999; Lara & Patton, 2000; Lara, Geise & Schneider, 2005; Clemente-Carvalho et al., 2009; Clemente-Carvalho et al., 2011).

We provide a description of the osteological traits of the three new species of *Chiasmocleis;* comparative data is not available for any of the species closely related to the new species. Furthermore, osteological data for *Chiasmocleis* is limited to: *C. albopunctata* (pectoral girdle, Parker, 1927; palatal region, Zweifel, 1986), *C. anatipes* (brief comments in Walker & Duellman, 1974), *C. avilapiresae* (brief comments in Peloso & Sturaro, 2008), *C. devriesi* (brief comments in Funk & Canatella, 2009), *C. gnoma* (pectoral girdle; Canedo, Dixo & Pombal, 2004), and *C. mehelyi* (pectoral girdle; Caramaschi & Cruz, 1997).

Among the new described species, *Chiasmocleis altomontana* exhibits the most ossified skull, e.g., no cartilage between prootic and exocippital bones, sphenethmoid fuse into a single element, more extensive ossification of the crista parotica, optic fenestra surrended entirely by bone, etc (Figs. 11 and 12). The heavy ossified skull easily distinguish this species from the other two new species.

The vomer is overall triangular in the new species and the posterior ramus does not contact with the sphenethmoid (Fig. 11). Only the vomer of *C. devriesi* has a posterior part reaching the sphenethmoid (Funk & Canatella, 2009). A posterior vomer fused with the sphenethmoid was reported for *Chiasmocleis anatipes* (Walker & Duellman, 1974) and *C. avilapiresae* (Peloso & Sturaro, 2008).

The extent of development of the sphenethmoid varies from paired to fused (Parker, 1934; Walker & Duellman, 1974; Lehr & Trueb, 2007; Peloso & Sturaro, 2008; Funk & Canatella, 2009; Morales & McDiarmid, 2009; Trueb Diaz & Blackburn, 2011). The sphenethmoids of *Chiasmocleis altomontana* are fused into a single and well-developed bone (Fig. 11); this contrast with reports for other small size *Chiasmocleis* (e.g., *C. antenori* Walker, 1973) where this bone is poorly developed.

*Chiasmocleis altomontana* exhibits a procoelous vertebral column, a condition also reported for *C. anatipes* (Walker & Duellman, 1974) and *C. avilapiresae* (Peloso & Sturaro, 2008). *Chiasmocleis albopunctata* (Parker, 1934) has a diplasiocoelous condition that is also observed in *Chiasmocleis migueli* and *C. veracruz*. Among the new species species, the pectoral girdle of *C*.* migueli* and *C. veracruz* have a cartilagious connection between the suprascapula and the coracoid, whereas in *C. altomontana* this area is fully ossified (Fig. 14).

The increase availability of morphological and molecular information for *Chiasmocleis* species will allow future phylogenetic analyses combining these data sets. In addition, it will help us to understand the evolution of osteological differences in a phylogenetic context and may provide additional phylogenetic hypotheses for species in the genus.

##  Supplemental Information

10.7717/peerj.3005/supp-1 Supplemental Information 1 Average genetic distances for 16S data used in the study

10.7717/peerj.3005/supp-2 Supplemental Information 2 Tree with posterior probabiities

10.7717/peerj.3005/supp-3 Supplemental Information 3 Concatenation Tree

10.7717/peerj.3005/supp-4 Supplemental Information 4 Tree showing quartet support

10.7717/peerj.3005/supp-5 Appendix I Specimens examined

10.7717/peerj.3005/supp-6 Appendix SII

## Additional Information and Declarations

### DNA Deposition

The following information was supplied regarding the deposition of DNA sequences: Genbank numbers are included in Appendix II along with other information for the samples.

### Data Availability

The following information was supplied regarding data availability: Genbank
KM111724–KM111726, KM111808–KM111816, KM111820–KM111822, KM111898–KM111907, KM111911–KM111913, KM111983–KM111994, KM112020, JQ410665–JQ410666, KY002063–KY002066 and KX809586–KX809591.

### New Species Registration

The following information was supplied regarding the registration of a newly described species: *Chiasmocleis migueli* sp. nov., urn:lsid:zoobank.org:act:C25510A7-8031-4EC6-B966-29F8A95DE8F6; *Chiasmocleis veracruz* sp. nov., urn:lsid:zoobank.org:act:528CB36C-871C-4A94-B524-E9C6B05734FD; *Chiasmocleis altomontana sp.* nov., urn:lsid:zoobank.org:act:5D2C0A91-9A1E-4379-A047-D4E967E69942; Publication LSID, urn:lsid:zoobank.org:pub:31554BA0-7A5D-4752-A9C2-F8A2978621B4
