# Supplementary material for: Molecular and morphological data reveal three new cryptic species of Chiasmocleis (Mehely 1904) (Anura, Microhylidae) endemic to the Atlantic Forest, Brazil
Source: PeerJ. 2017 Feb 21;5:e3005. doi: 10.7717/peerj.3005 (PMC5322761; doi:10.7717/peerj.3005)
Supplement: Appendix I [file peerj-05-3005-s005.docx]

**APPENDIX I**

*Chiasmocleis alagoana:*Brazil: Alagoas (MNRJ 21856, holotype, MNRJ 21857, 21859 paratypes)

*Chiasmocleis atlantica:* Brazil: Rio de Janeiro: Nova Iguaçu (MNRJ 17551–54 paratype); São Paulo: Ubatuba (MZUSP 70745, 134117)

*Chiasmocleis lacrimae:* Brazil: Espirito Santo: Guarapari (MZUSP 13159); Rio de Janeiro: Cachoeira do Macacu (MNRJ 38105), Duque de Caxias (MNRJ 17566, 17568), Horto Florestal, Seropédica Itaguaí (MZUSP 82529–32, 82852–58; MNRJ17480–82, 17485–86, 17488–90, 17492, 17498–99, 17502–03 Paratype), Ilha de Cabo Frio, Arraial do Cabo (MNRJ 17555), Macaé (MNRJ 47377), Mimoso do Sul (MNRJ 66495); São Paulo, Bertioga (MZUSP 136256), Picinguava, Ubatuba (CFBH 4270, 4046, 4051, 11999, 12003, 12955).

*Chiasmocleis capixaba:*Brazil: Bahia: Nova Viçosa (MNRJ 18924); Espirito Santo: Araracruz (MNRJ 17515–29; CFBH 2668–69, 2701–02 paratypes), Guarapari (MZUSP 142952–69).

*Chiasmocleis cordeiroi*: Brazil: Bahia: Amargosa (UFBA 2328); Projeto de Assentamento Zumbi dos Palmares, Camamu (MNRJ 29931 holotype); Igrapiúna (MZUESC 9086–87, 9286–89); Itacaré (MNRJ 35364, 35382–83); E.E. Wenceslau Guimarães, Wenceslau Guimarães (Field number MRT 22117–25).

*Chiasmocleis crucis*: Brazil: Bahia: Projeto de Assentamento Zumbi dos Palmares, Camamu (MNRJ 2993 Holotype; MNRJ 29936–37 paratypes); RPPN Serra Bonita, Camacan (MZUESC 9028–29, 9031–34, 9036, MZUSP 147408; Field number MRT 15920, 15935, 15940, 16069–71, 16106, 16118, 16198, 16987); Ilhéus, Mata UESC (MZUSP 147403–04), Ponta da Tulia (MZUSP147405–07); Una (MNRJ 28585, 28591, 28595–96, 28600; Field number MRT 5831).

*Chiasmocleis gnoma:* Brazil: Bahia: Estação Biológica de Una, Una (MNRJ 28625–27, 28629–41 paratypes), Porto Seguro (MZUSP 126382).

*Chiasmocleis leucosticta:*Brazil: Paraná, Morretes (MHNCI 1594–98), Paranagua (MHNCI 768–69); Santa Catarina: Córrego Grande, Florianópolis (MZUSP 139417–23); São Paulo: Cananéia (CFBH 5237), Eldorado Paulista (MZUSP 135620–22, 135634–44), Ilha Bela (MNRJ 23663, 9033), MZUSP Parque Estadual Carlos Botelho, São Miguel Arcanjo(MZUSP 136050–59).

*Chiasmocleis mantiqueira:*Brazil: Minas Gerais: Parque Estadual da Serra do Brigadeiro, Ervália (MNRJ 43404–06, 43408–15 paratypes), Rio Branco (UFMG 9640–41 9650–51, 9653–56, 9658–59); São Paulo: Piquete (CFBH 24128–34).

*Chiasmocleis schubarti:* Brazil: Bahia, Porto Seguro (MNRJ 38864, 27256); Espirito Santo: Soretama (MZUSP 82507–11, 85236, 85294, 85308–54); Minas Gerais: Bom Jesus do Galho (UFMG 3358); Morro do Pilar e Conceição do Mato Dentro (MCNAM 15026); Paruqe Estadual do Rio Doce (MZUFV 2602, 2605–06, 2610, 2613, 2615–16, 2628, 2649, 2787, 3411); Rio Piracicaba (MCNAM 14988); São Gonçalo do Rio Abaixo (MCNAM 13725 –32);

*Chiasmocleis sapiranga:* Brazil: Bahia: Reserva Sapiranga, Mata de São João (UFBA 4986 Holotype, UFBA 4981, 4983–84, MNRJ 41851paratypes).
