# Supplementary material for: Molecular and morphological data reveal three new cryptic species of Chiasmocleis (Mehely 1904) (Anura, Microhylidae) endemic to the Atlantic Forest, Brazil
Source: PeerJ. 2017 Feb 21;5:e3005. doi: 10.7717/peerj.3005 (PMC5322761; doi:10.7717/peerj.3005)
Supplement: Appendix SII [file peerj-05-3005-s006.pdf]

Chiasmocleis	Voucher	Tissue	Locality
alagoanus		MNH-UFAL2682	
alagoanus		MNH-UFAL2683	
altomontana sp.nov	MZUSP133641	CTMZ1147	Estação Ecológica do Bananal
altomontana sp.nov	MZUSP133640	CTMZ1172	Estação Ecológica do Bananal
capixaba	MZUSP147497	CTA1861	ReBio de Duas Bocas
capixaba	MZUSP147498	CTA1863	ReBio de Duas Bocas
capixaba	MZUSP147499	CTA1864	ReBio de Duas Bocas
capixaba	MZUSP147468	CTA1865	ReBio de Duas Bocas
capixaba	MZUSP147482	CTA1869	ReBio de Duas Bocas
capixaba	MZUSP147469	CTA1870	ReBio de Duas Bocas
capixaba	MZUSP147500	CTA1871	Costa Bela
capixaba	MZUSP147510	CTA1872	Costa Bela
capixaba	MZUSP147512	CTA1874	Costa Bela
capixaba	MZUSP147513	CTA1875	Costa Bela
capixaba	JFT479	CTA1876	Costa Bela
capixaba	MZUSP147514	CTA1877	Costa Bela
capixaba	JFT483	CTA1879	Costa Bela
capixaba	JFT499	CTA1881	Costa Bela
capixaba	MZUSP147520	CTA1893	Costa Bela
capixaba	MZUSP147521	CTA1894	Costa Bela
capixaba	MZUSP147523	CTA1896	Costa Bela
capixaba	MTR12276	CTMZ6907	FLONA de Goytacazes
capixaba	MTR12296	CTMZ6908	FLONA de Goytacazes
capixaba	MTR12407	CTMZ6912	Reserva Vale do Rio Doce
capixaba	MTR12484	CTMZ6920	Reserva Vale do Rio Doce
capixaba	MTR12485	CTMZ6921	Reserva Vale do Rio Doce
capixaba	-	CTMZ6939	-
capixaba	MTR12076	MTR12076	Reserva Vale do Rio Doce
capixaba	MTR12297	MTR12297	FLONA de Goytacazes
cordeiroi	CFBH32057	CFBH15784	Road conecting Ilhéus to Itacaré
cordeiroi	MZUSP147496	CTA1935	Reserva Ecológica da Michelin
cordeiroi	MTR22122	MTR22122	Estação Ecológica Wenceslau Guimar
cordeiroi	MTR22123	MTR22123	Estação Ecológica Wenceslau Guimar
cordeiroi	PEU137	PEU137	-
cordeiroi	PEU146	PEU146	-
crucis	MTR6001	CTMZ6898	Serrado Teimoso
crucis	-	CTMZ6900	Estação Experimental Almada
crucis	-	CTMZ6901	Estação Experimental Almada
crucis	MTR16070	MTR16070	Serra Bonita
lacrimae	-	CFBH73	Picinguaba
lacrimae	CFBH17495	CFBH7361	Ilha de São Sebastião
lacrimae	-	CFBH76	Picinguaba
lacrimae	JFT981	CTA1934	Usina Paineiras
lacrimae	-	CTMZ6946	

lacrimae	RN7003	CTRN173	Ilha Grande
lacrimae	RN7004	CTRN174	Ilha Grande
lacrimae	RN7005	CTRN175	Ilha Grande
lacrimae	MNRJ47477	MNRJ47477	ReBio União
lacrimae	MNRJ48415	MNRJ48415	ReBio União
lacrimae	MNRJ49302	MNRJ49302	
lacrimae	MNRJ60744	MNRJ60744	
lacrimae	MNRJ66494	MNRJ66494	
leucosticta	CFBH19029	CFBH8594	Parque Estadual da Ilha do Cardoso
leucosticta	MZUSP136053	CTMZ2485	Parque Estadual de Carlos Botelho
leucosticta	MZUSP136055	CTMZ2493	Parque Estadual de Carlos Botelho
leucosticta	MZUSP136059	CTMZ2497	Parque Estadual de Carlos Botelho
leucosticta	MTR7128	CTMZ6943	Parque Estadual Intervales
leucosticta	-	CTMZ6944	-
mantiqueira	-	CTMZ6891	Serra do Brigadeiro
mantiqueira	UFMG-A9643	UFMG-T1802	-
mantiqueira	UFMG-A9659	UFMG-T1804	-
mantiqueira	UFMG-A9651	UFMG-T1810	-
mantiqueira	UFMG-A9656	UFMG-T1815	-
quilombola	-	CFBH1437	ReBio de Sooretama
quilombola	-	CFBH1438	ReBio de Sooretama
quilombola	CFBH19471	CFBH9055	Plantação de Cacau - Povoação
quilombola	CFBH18076	CFBH9082	Plantação de Cacau - Povoação
quilombola	CFBH18077	CFBH9083	Plantação de Cacau - Povoação
quilombola	JFT831	CTA1906	FLONA do Rio Preto
quilombola	MZUSP147471	CTA1907	FLONA do Rio Preto
quilombola	MZUSP147472	CTA1908	FLONA do Rio Preto
quilombola	MZUSP147473	CTA1909	FLONA do Rio Preto
quilombola	MZUSP147474	CTA1918	FLONA do Rio Preto
quilombola	MZUSP147475	CTA1919	FLONA do Rio Preto
quilombola	MZUSP147494	CTA1923	FLONA do Rio Preto
quilombola	MZUSP147479	CTA1929	FLONA do Rio Preto
quilombola	MZUSP147480	CTA1931	FLONA do Rio Preto
quilombola	MZUSP147493	CTA1933	FLONA do Rio Preto
quilombola	JFT990	CTA1938	Parque Estadual de Itaúnas
quilombola	MTR12017	CTMZ6903	Reserva Vale do Rio Doce
quilombola	MTR12077	CTMZ6905	Reserva Vale do Rio Doce
quilombola	MTR12470	CTMZ6916	Reserva Vale do Rio Doce
quilombola	MTR12471	CTMZ6917	Reserva Vale do Rio Doce
quilombola	MTR21527	LGA3267	ReBio do Córrego Veado
schubarti	CFBH9331	CFBH2078	ReBio de Sooretama
schubarti	CFBH18075	CFBH9060	Plantação de Cacau - Povoação
schubarti	CFBH 22501	CTA1860	ReBio de Duas Bocas
schubarti	MZUSP147485	CTA1887	FLONA do Rio Preto
schubarti	MZUSP147487	CTA1924	FLONA do Rio Preto

schubarti	MTR12094	CTMZ6906	FLONA de Goytacazes
schubarti	LGA2630	LGA2630	ReBio do Córrego Veado
schubarti	MTR12266	MTR12266	FLONA de Goytacazes
schubarti	MTR17524	MTR17524	Parque Estadual do Rio Doce
schubarti	MTR17571	MTR17571	Parque Estadual do Rio Doce
shudikarensis	MW5645	-	sample from French Guiana
veracruz sp. nov.	-	CFBH15818	Estação Ecológica da Veracel
veracruz sp. nov.	MTR13466	CTMZ6923	Fazenda Nova Alegria
veracruz sp. nov.	MTR13489	CTMZ6924	Fazenda Nova Alegria
veracruz sp. nov.	MTR13495	CTMZ6925	Fazenda Nova Alegria
veracruz sp. nov.	MTR13545	CTMZ6927	Fazenda Nova Alegria
veracruz sp. nov.	MTR13547	CTMZ6929	Fazenda Nova Alegria
veracruz sp. nov.	MTR13548	CTMZ6930	Fazenda Nova Alegria
veracruz sp. nov.	MTR13565	CTMZ6932	Fazenda Nova Alegria
veracruz sp. nov.	MTR13579	CTMZ6933	Fazenda Nova Alegria
veracruz sp. nov.	MTR13589	CTMZ6935	Fazenda Nova Alegria

Municipality	State	12S	16S	ND2
Maceio	Alogoas			
Maceio	Alogoas			
	São Paulo	KX809586	KX809588	-
	São Paulo	KX809587	KX809589	-
Cariacica	Espírito Santo	KM111721	KM111817	JQ410706
Cariacica	Espírito Santo	KM111722	KM111818	JQ410707
Cariacica	Espírito Santo	KM111723	KM111819	JQ410708
Cariacica	Espírito Santo	KM111724	KM111820	KM111992
Cariacica	Espírito Santo	KM111725	KM111821	KM111993
Cariacica	Espírito Santo	KM111726	KM111822	KM111994
Serra	Espírito Santo	KM111727	KM111823	JQ410709
Serra	Espírito Santo	KM111728	KM111824	JQ410685
Serra	Espírito Santo	KM111729	KM111825	JQ410690
Serra	Espírito Santo	KM111730	KM111826	JQ410691
Serra	Espírito Santo	KM111731	KM111827	JQ410692
Serra	Espírito Santo	KM111732	KM111828	JQ410693
Serra	Espírito Santo	KM111733	KM111829	JQ410694
Serra	Espírito Santo	KM111734	KM111830	JQ410695
Serra	Espírito Santo	KM111735	KM111831	JQ410700
Serra	Espírito Santo	KM111736	KM111832	JQ410701
Serra	Espírito Santo	KM111737	KM111833	JQ410702
Linhares	Espírito Santo	KM111738	KM111834	-
Linhares	Espírito Santo	KM111739	KM111835	JQ410688
Linhares	Espírito Santo	KM111740	KM111836	-
Linhares	Espírito Santo	KM111741	KM111837	-
Linhares	Espírito Santo	KM111742	KM111838	-
Guarapari	Espírito Santo	KM111743	KM111839	-
Linhares	Espírito Santo	KM111744	KM111840	JQ410687
Linhares	Espírito Santo	KM111745	KM111841	JQ410689
Ilhéus	Bahia	KM111759	KM111852	KM111995
Ituberá	Bahia	KM111760	KM111853	KM111996
Wenceslau Guimarãe	Bahia	KM111761	KM111854	KM111997
Wenceslau Guimarãe	Bahia	KM111762	KM111855	KM111998
Jaguaripe	Bahia	KM111763	KM111856	KM111999
Jaguaripe	Bahia	KM111764	KM111857	KM112000
Jussari	Bahia	KM111765	KM111858	KM112001
Ilhéus	Bahia	KM111766	KM111859	KM112002
Ilhéus	Bahia	KM111767	KM111860	KM112003
Camacan	Bahia	KM111768	KM111861	KM112004
Ubatuba	São Paulo	KM111748	KC180040	JQ410715
Ilha Bela	São Paulo	KM111749	KM111844	-
Ubatuba	São Paulo	KM111750	KC180063	JQ410714
Itapemirim	Espírito Santo	KM111751	KM111845	JQ410710
Bertioga	São Paulo	KM111752	KM111846	-

Angra dos Reis	Rio de Janeiro	KM111753	KM111847	-
Angra dos Reis	Rio de Janeiro	KM111754	KM111848	-
Angra dos Reis	Rio de Janeiro	KM111755	KM111849	-
Macaé	Rio de Janeiro	KM111746	KM111842	-
Macaé	Rio de Janeiro	KM111747	KM111843	-
Cachoeiras de Maca	Rio de Janeiro	KM111756	KM111850	JQ410712
Duque de Caxias	Rio de Janeiro	KM111757	-	JQ410713
Mimoso do Sul	Espírito Santo	KM111758	KM111851	JQ410711
Cananéia	São Paulo	KM111769	KM111862	-
São Miguel Arcanjo	São Paulo	KM111770	KM111863	-
São Miguel Arcanjo	São Paulo	KM111771	-	-
São Miguel Arcanjo	São Paulo	-	KM111864	-
Ribeirão Grande	São Paulo	-	-	KM112005
Piedade	São Paulo	KM111772	-	KM112006
Araponga	Minas Gerais	KM111773	KM111865	-
Ouro Branco	Minas Gerais	KM111774	KM111866	KM112007
Ouro Branco	Minas Gerais	KM111775	KM111867	-
Ouro Branco	Minas Gerais	KM111776	KM111868	-
Ouro Branco	Minas Gerais	KM111777	KM111869	-
Linhares	Espírito Santo	KM111778	KC180044	-
Linhares	Espírito Santo	KM111779	KC179977	-
Linhares	Espírito Santo	KM111780	KM111870	KM112008
Linhares	Espírito Santo	KM111781	KM111871	KM112009
Linhares	Espírito Santo	KM111782	KM111872	KM112010
Conceição da Barra	Espírito Santo	KM111783	KM111873	JQ410669
Conceição da Barra	Espírito Santo	KM111784	KM111874	JQ410670
Conceição da Barra	Espírito Santo	KM111785	KM111875	JQ410671
Conceição da Barra	Espírito Santo	KM111786	KM111876	JQ410672
Conceição da Barra	Espírito Santo	KM111787	KM111877	JQ410673
Conceição da Barra	Espírito Santo	KM111788	KM111878	JQ410674
Conceição da Barra	Espírito Santo	KM111789	KM111879	JQ410677
Conceição da Barra	Espírito Santo	KM111790	KM111880	JQ410679
Conceição da Barra	Espírito Santo	KM111791	KM111881	JQ410680
Conceição da Barra	Espírito Santo	KM111792	KM111882	JQ410681
Conceição da Barra	Espírito Santo	-	KM111883	-
Linhares	Espírito Santo	KM111793	KM111884	-
Linhares	Espírito Santo	KM111794	KM111885	-
Linhares	Espírito Santo	KM111795	KM111886	-
Linhares	Espírito Santo	KM111796	KM111887	-
Pinheiros	Espírito Santo	KM111797	KM111888	JQ410668
Linhares	Espírito Santo	KM111798	KC180071	KM112011
Linhares	Espírito Santo	KM111799	KM111889	KM112012
Cariacica	Espírito Santo	KM111800	KM111890	KM112013
Conceição da Barra	Espírito Santo	KM111801	KM111891	KM112014
Conceição da Barra	Espírito Santo	KM111802	KM111892	KM112015

Linhares	Espírito Santo	KM111803	KM111893	KM112016
Pinheiros	Espírito Santo	KM111804	KM111894	JQ410661
Linhares	Espírito Santo	KM111805	KM111895	KM112017
Marliéria	Minas Gerais	KM111806	KM111896	KM112018
Marliéria	Minas Gerais	KM111807	KM111897	KM112019
		KY002063	KY002064	KY002066
Porto Seguro	Bahia	-	KM111898	-
Trancoso	Bahia	KM111808	KM111899	JQ410665
Trancoso	Bahia	KM111809	KM111900	JQ410666
Trancoso	Bahia	KM111810	KM111901	-
Trancoso	Bahia	KM111811	KM111902	-
Trancoso	Bahia	KM111812	KM111903	-
Trancoso	Bahia	KM111813	KM111904	-
Trancoso	Bahia	KM111814	KM111905	-
Trancoso	Bahia	KM111815	KM111906	-
Trancoso	Bahia	KM111816	KM111907	KM112020

BDNF
KX809590
KX809591
KM111908
KM111909
KM111910
KM111911
KM111912
KM111913
KM111914
KM111915
KM111916
KM111917
KM111918
KM111919
KM111920
KM111921
KM111922
KM111923
KM111924
KM111925
KM111926
-
KM111927
KM111928
KM111929
KM111930
KM111931
KM111939
KM111940
KM111941
KM111942
KM111943
KM111944
KM111945
KM111946
KM111947
KM111948
KC180202
KC180163
KM111932
KM111933

-
KM111934
KM111935
-
-
KM111936
KM111937
KM111938
-
-
KM111949
KM111950
KM111951
-
KM111952
-
KM111953
KM111954
KM111955
KC180193
KC180168
KM111956
KM111957
KM111958
KM111959
KM111960
KM111961
KM111962
KM111963
KM111964
KM111965
KM111966
KM111967
KM111968
KM111969
KM111970
-
KM111971
KM111972
KM111973
KC180122
KM111974
KM111975
KM111976
KM111977

KM111978
KM111979
KM111980
KM111981
KM111982
KY002065
KM111983
KM111984
KM111985
KM111986
KM111987
KM111988
KM111989
KM111990
KM111991
-
